# Sustained HIV-1 remission after heterozygous CCR5Δ32 stem cell transplantation

**DOI:** 10.1038/s41586-025-09893-0

**Published:** 2025-12-01

**Authors:** Christian Gaebler, Samad Kor, Kristina Allers, Michela Perotti, David Mwangi, Karolin Meixenberger, Kirsten Hanke, Timo Trenkner, Tom Kraus, Yeqin Sha, Carmen Arentowicz, Stanley Odidika, Nikolai Grahn, Rachel Scheck, Naomi Perkins, Marion Pardons, Vanessa Igbokwe, Victor Corman, Thomas Burmeister, Olga Blau, Gülüstan Sürücü, Axel Pruß, Christian G. Schneider, Gerd Klausen, Jürgen Sauter, Florian Klein, Leif E. Sander, Jörg Hofmann, Lam Vuong, Lars Bullinger, Livius Penter, Henning Gruell, Daniel B. Reeves, Philipp Schommers, Angelique Hoelzemer, Martin Obermeier, Igor W. Blau, Thomas Schneider, Olaf Penack

**Affiliations:** 1 Laboratory of Translational Immunology of Viral Infections, Department of Infectious Diseases and Critical Care Medicine, Charité–Universitätsmedizin Berlin, Corporate Member of Freie Universität Berlin and Humboldt-Universität zu Berlin, Berlin, Germany; 2 Berlin Institute of Health, Berlin, Germany; 3 Department of Hematology, Oncology and Tumor Immunology, Charité–Universitätsmedizin Berlin, Corporate Member of Freie Universität Berlin and Humboldt-Universität zu Berlin, Berlin, Germany; 4 National Center for Tumor Diseases (NCT), Partner Site, Berlin, Germany; 5 Department of Gastroenterology, Infectious Diseases and Rheumatology, Charité–Universitätsmedizin Berlin, Corporate Member of Freie Universität Berlin and Humboldt-Universität zu Berlin, Berlin, Germany; 6 Laboratory MVZ MIB–Medical Center for Infectious Diseases, Berlin, Germany; 7 Division of Sexually Transmitted Bacterial Pathogens (STI) and HIV, Robert Koch Institute, Berlin, Germany; 8 First Department of Medicine, University Medical Center Hamburg-Eppendorf, Hamburg, Germany; 9 Department I of Internal Medicine, Faculty of Medicine and University Hospital Cologne, University of Cologne, Cologne, Germany; 10 German Center for Infection Research (DZIF), Partner Site Bonn-Cologne, Cologne, Germany; 11 Center for Molecular Medicine Cologne (CMMC), University of Cologne, Cologne, Germany; 12 Institute of Virology, Charité–Universitätsmedizin Berlin, Corporate Member of Freie Universität Berlin and Humboldt-Universität zu Berlin, Berlin, Germany; 13 Labor Berlin-Charité Vivantes, Berlin, Germany; 14 Institute of Transfusion Medicine, Charité–Universitätsmedizin Berlin, Corporate Member of Freie Universität Berlin and Humboldt-Universität zu Berlin, Berlin, Germany; 15 SPP-Mitte, Specialist Practice for Infection Medicine at Oranienburger Tor, Berlin, Germany; 16 DKMS Group, Tübingen, Germany; 17 Laboratory of Experimental Immunology, Institute of Virology, Faculty of Medicine and University Hospital Cologne, University of Cologne, Cologne, Germany; 18 Department of Infectious Diseases and Critical Care Medicine, Charité–Universitätsmedizin Berlin, Corporate Member of Freie Universität Berlin and Humboldt-Universität zu Berlin, Berlin, Germany; 19 Vaccine and Infectious Disease Division, Fred Hutchinson Cancer Center, Seattle, WA USA; 20 Leibniz Institute of Virology, Hamburg, Germany; 21 Institute for Infection and Vaccine Development (IIRVD), University Medical Center Hamburg-Eppendorf, Hamburg, Germany

**Keywords:** Stem-cell research, Viral infection

## Abstract

HIV cure is exceptionally rare, with only six cases documented among the estimated 88 million individuals who have acquired HIV since the onset of the epidemic^1–6^. Successful cures, including that of the pioneering individual known as the Berlin patient, are limited to those who received allogeneic stem cell transplants (allo-SCTs) for haematological cancers. HIV resistance from stem cell donors with the rare homozygous CCR5Δ32 mutation was long considered the main mechanism for HIV remission without antiretroviral therapy. However, recent reports have highlighted CCR5-independent mechanisms as important contributors to HIV cure^6–8^. Here we provide new evidence for this conceptual shift, whereby long, treatment-free HIV remission was achieved after allo-SCT with functionally active CCR5. A man with heterozygous CCR5 wild-type/Δ32 living with HIV received allo-SCT from a HLA-matched unrelated heterozygous CCR5 wild-type/Δ32 donor as treatment for acute myeloid leukaemia. Three years after allo-SCT, the patient discontinued antiretroviral therapy. So far, HIV remission has been sustained for more than 6 years with undetectable plasma HIV RNA. Reservoir analysis revealed intact proviral HIV before transplantation, but no replication-competent virus in blood or intestinal tissues after allo-SCT. Declining or absent HIV-specific antibody and T cell responses support the absence of viral activity. High antibody-dependent cellular cytotoxicity activity at the time of transplantation may have contributed to HIV reservoir clearance. These results demonstrate that CCR5Δ32-mediated HIV resistance is not essential for durable remission, which underscores the importance of effective viral reservoir reductions in HIV cure strategies.

## Abstract (web summary)

HIV remission of more than 6 years was achieved in a patient with functional viral co-receptors after CCR5 wild-type/Δ32 allogeneic stem cell transplantation, providing evidence of other mechanisms that can be harnessed to attain long-term remission.

## Main

Antiretroviral therapy (ART) is highly effective in suppressing HIV replication. However, when ART is discontinued, rapid viral rebound occurs within 4 weeks in most individuals living with HIV^9^. Consequently, lifelong therapy is required to prevent viral reactivation and disease progression to immunodeficiency. Although AIDS-related deaths are declining, more than 1 million new HIV acquisitions occur each year. As a result, the number of individuals living with HIV requiring chronic treatment is projected to increase to 46 million by 2050 (ref. ^10^). Therefore, the search for a curative intervention that enables individuals living with HIV to safely stop therapy and live long, healthy lives is a global research priority^11^. Yet, more than 40 years into the HIV pandemic, a scalable cure for HIV remains elusive. Most reported cases involve individuals undergoing allo-SCT from CCR5Δ32/Δ32 homozygous donors, whose cells lack the essential viral co-receptor CCR5, which confers resistance to HIV infection^1–5^. Transplants without the homozygous CCR5Δ32 mutation were previously thought to be ineffective for sustaining HIV remission without ART beyond 9 months^12,13^. Recently, reports from a large cohort study^7^, a non-human primate model^8^ and the case of the individual known as the Geneva patient^6^ demonstrated substantial reductions in persistent HIV reservoirs and viral suppression surpassing 2.5 years after allo-SCT with wild-type CCR5 donor cells. These results highlight CCR5-independent mechanisms as important contributors to achieving HIV remission.

## Clinical course

**Fig. 1:**
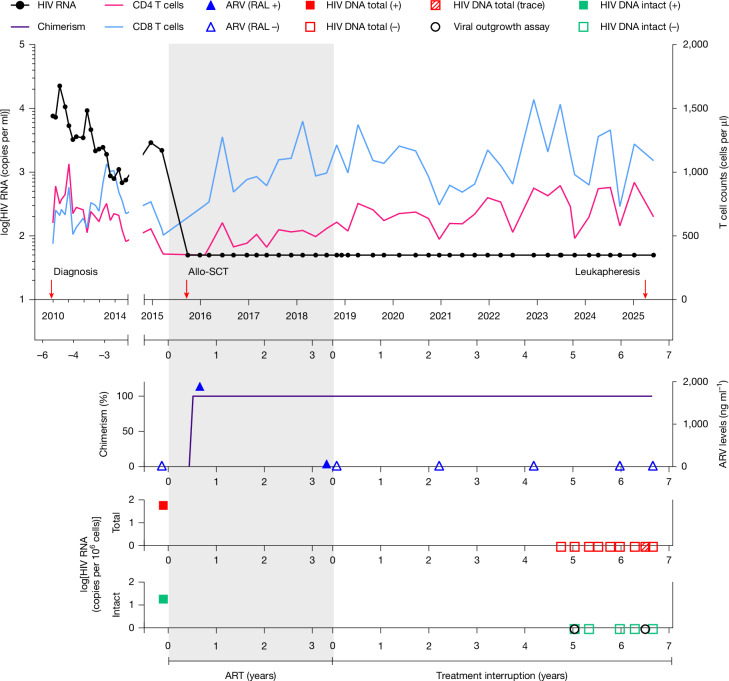
Longitudinal follow-up. Top, timeline of plasma HIV-1 RNA levels (black line; left* y* axis) and T cell counts (CD4, pink; CD8, blue; right *y* axis) from HIV diagnosis until last follow-up. Red arrows indicate the time of HIV diagnosis, allo-SCT and leukapheresis. Middle, donor chimerism (per cent donor cells; purple). The grey shaded area indicates time on ART (raltegravir (RAL), ABC/3TC). Blue filled and open triangles indicate time points with or without detectable antiretroviral (ARV) drug levels (that is, RAL), respectively (right *y* axis). Bottom, red and green squares indicate time points with detectable total or intact HIV proviral DNA, respectively (left *y* axis). Undetectable or traces of HIV proviral DNA concentrations are represented by open or scattered squares, respectively. Open black circles indicate time points with undetectable replication competent HIV measured by viral outgrowth assays.

Here we report a 60-year-old man, referred to as the second Berlin patient (B2), who was diagnosed with HIV-1 subtype B in December 2009. At the time of diagnosis, the individual presented with a CD4 T cell count of 602 cells per µl and a HIV-1 plasma viral load of 7,600 copies per ml. As a participant of the Strategic Timing of Antiretroviral Treatment (STAR) trial^14^, he did not receive immediate ART and remained in good health without HIV-related symptoms for 5 years. During this initial period without ART, the highest HIV-1 plasma viral load was 22,500 copies per ml, with a CD4 nadir of 358 cells per µl. Overall, patient B2 showed a favourable clinical course, with viral set points around 2,000 copies per ml and CD4 counts above 500 cells per µl for most of this period (Fig. 1 and Supplementary Table 1).

In April 2015, the patient’s condition deteriorated, and he was subsequently diagnosed with acute myeloid leukaemia (AML): 46, XY, with molecular aberrations in the tyrosine kinase domain of FLT3. He received two cycles of AML induction chemotherapy that contained cytarabine and daunorubicin and achieved complete haematological remission. In the subsequent months, two additional consolidation cycles of high-dose cytarabine were administered, concluding in August 2015. In parallel to AML therapy, ART was initiated with raltegravir and abacavir–lamivudine (ABC/3TC) (Fig. 1).

**Extended Data Fig. 1:**
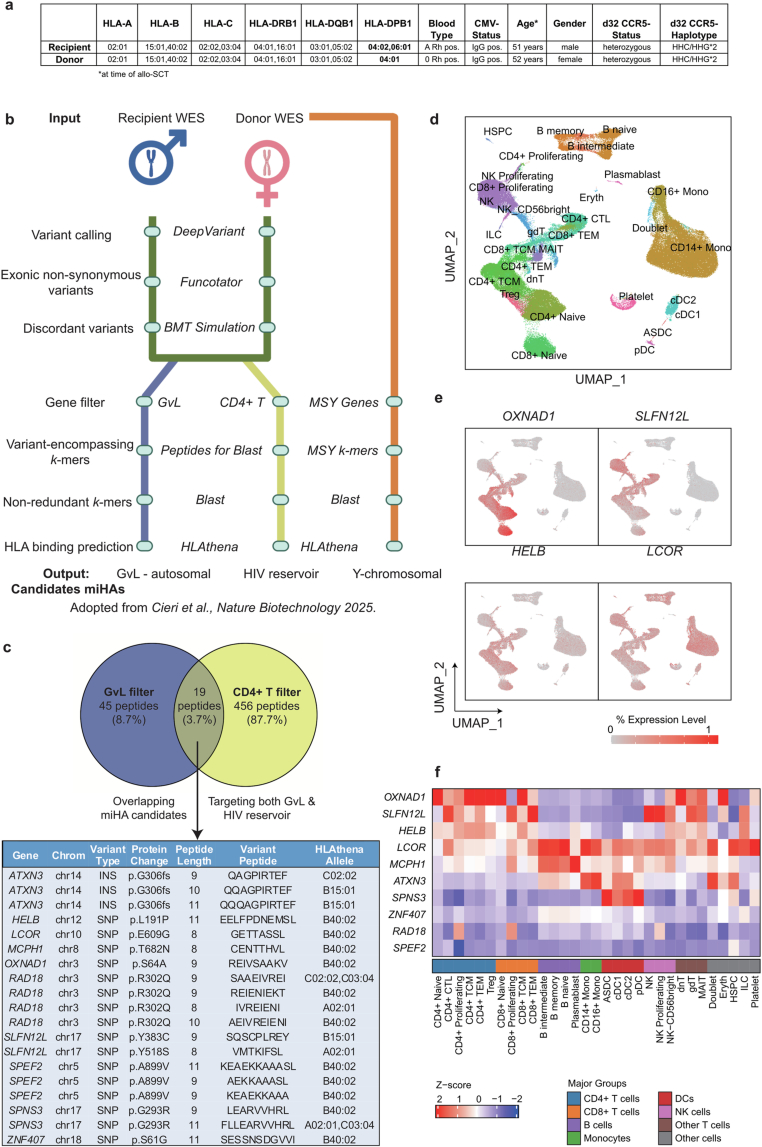
Minor histocompatibility antigen (miHA) identification. **a**, Recipient and donor characteristics. **b**, Overview of the analysis workflow for miHA prediction^18^ (BMT, bone marrow transplantation; MSY, male-specific Y chromosome region) **c**, Venn diagram of 64 potential autosomal GVL-mediating miHAs and 475 potential CD4 T cell-related miHAs, with 19 peptides overlapping. The table below details the overlapping peptides. **d**, UMAP representation of cell type annotations of public PBMC datasets used to generate the CD4 T cell-related gene filter. **e**, UMAP representations of genes encoding miHA candidates that are predicted to drive responses against GVL and the HIV reservoir **f**, Heatmap of 10 genes encoding 19 overlapping miHAs across different cell types in PBMC. Analysis workflow for miHA prediction in **b** adapted from ref. ^18^, Springer Nature America, using BioRender. Penter, L. (2025) https://BioRender.com/r9akwx6.

**Extended Data Fig. 2:**
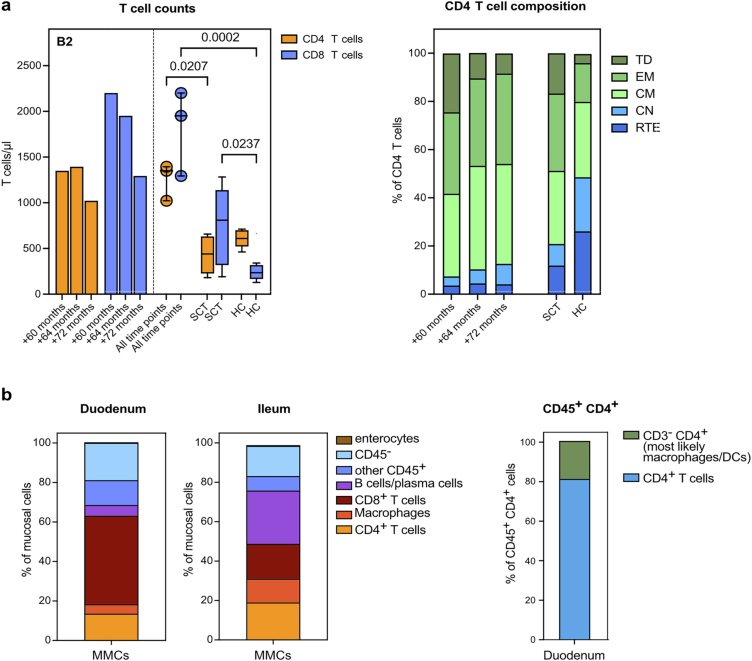
T cell composition in peripheral blood and Lamina Propria Leucocytes after allo-SCT. **a**, Peripheral CD4 and CD8 T cell counts and distribution of terminally differentiated (TD), effector memory (EM), central memory (CM), central naïve (CN) cells, and recent thymic emigrants (RTE) in CD4 T cells from patient B2 at different time points after allo-SCT compared to HIV-negative control individuals without (HC, n = 10) or with (SCT, n = 6) allo-SCT. Aligned dot plots show individual data points with the median value indicated by a horizontal line. Error bars represent the interquartile range (IQR), spanning from the 25^th^ to the 75^th^ percentile. Box- and whisker plots represent the IQR from the 25^th^ and 75^th^ percentile, with the line within the box indicating the median value. Whiskers extend to the minimum and maximum values within 1.5 times the IQR from the lower and upper quartiles. Statistical significance was determined using the Kruskal-Wallis test followed by Dunn’s post-hoc test. A two-sided test was applied, and no adjustment were made for multiple comparisons. **b**, Cell populations in mucosal mononuclear cell (MMC) suspensions obtained from the duodenum or ileum and composition of CD45+CD4+ cells sorted from duodenal mononuclear cells.

In the AML treatment course, an allo-SCT was planned. We first tested family members and identified a 10/10 HLA-matched sibling donor (no mismatches in the HLA loci A, B, C, DR or DQ) with wild-type CCR5 status. We then performed a search in the German Stem Cell Donor Registry (DKMS) and identified several 10/10 HLA-matched unrelated stem cell donors. We did not identify a homozygous CCR5 Δ32/Δ32 donor; however, an unrelated 10/10 HLA-matched woman had a CCR5 wild-type/Δ32 heterozygous genotype. Notably, patient B2 was also heterozygous for CCR5Δ32 (Extended Data Fig. 1a). After consultation with the patient, we selected this CCR5 wild-type/Δ32 donor for allo-SCT. This decision was based on the following criteria: (1) comparable allo-SCT outcomes between matched-related and matched-unrelated donors^15^; and (2) potential clinical advantages in long-term HIV management mediated by the CCR5 heterozygous genotype of the unrelated donor compared with the CCR5 wild-type genotype of the sibling donor^16^. The patient, who was 51 years old at that time, received the allo-SCT from peripheral blood stem cells in October 2015. Myeloablative reduced-intensity conditioning was performed with 8 Gy total body irradiation and fludarabine. Standard graft-versus-host disease (GVHD) prophylaxis was performed with cyclosporine, short-course methotrexate and anti-thymocyte globulin. Allo-SCT was well tolerated without major complications, except for the presence of grade I acute GVHD (skin only), which resolved with topical steroids. Consistent with this observation, high-resolution HLA class II typing identified a donor–recipient HLA-DPB1 mismatch classified as non-permissive in the graft-versus-host direction according to the DPB1 T cell epitope algorithm^17^ (Extended Data Fig. 1a). Furthermore, immunogenetic profiling of the donor and recipient through germline whole-exome sequencing and subsequent minor histocompatibility antigen (miHA) prediction^18^ revealed 64 potential graft-versus-leukaemia (GVL)-mediating peptides, including 19 from 10 autosomal genes expressed in CD4 T cells and 137 miHA candidates arising from Y chromosomal transcripts (Extended Data Fig. 1b–f and Supplementary Tables 2–4). Donor stem cells were successfully engrafted, and complete donor chimerism in peripheral blood was achieved in the first control on day +28 after transplantation and has been maintained ever since (Fig. 1). Adequate reconstitution of T cell subsets was observed, with elevated CD8 T cell counts commonly seen in people living with HIV after allo-SCT^19^ (Fig. 1 and Extended Data Fig. 2a). The AML remains in complete remission. ART was continued after allo-SCT until the patient independently decided to discontinue ART in September 2018. Analyses of plasma viral load were performed on a regular basis thereafter (treatment interruption; Fig. 1). Plasma concentrations of raltegravir were detected by liquid chromatography with tandem mass spectrometry (LC–MS/MS) during ART. No antiretroviral drugs were detected before initiation and after the interruption of ART (Fig. 1 and Extended Data Table 1). After 6 years, the patient remains in HIV remission with undetectable HIV-1 plasma viral loads (limit of detection of 25 or 14 copies per ml; Methods).

## HIV reservoir

Total HIV-1 DNA was readily detected in buffy coat cells collected before allo-SCT and ART initiation (59.9 and 70.0 copies per million cells, respectively) (Fig. 1 and Extended Data Table 2). These frequencies were similar to those reported in a large cohort of people living with HIV^20^. At the same time point, 23% of detected proviruses were classified as intact (15.97 copies per million cells) using an adapted intact proviral DNA assay (IPDA)^21,22^. By contrast, no intact HIV DNA was detected 5 years after treatment interruption in repeated measurements after allo-SCT.Measurements included proviral DNA, IPDA, Q4 droplet digital PCR (Q4ddPCR, Methods) and viral outgrowth assay quantification in peripheral blood mononuclear cells (PBMCs) and CD4 T cells. Assessments of mucosal mononuclear cells isolated from duodenal and ileal biopsies obtained during an intestinal endoscopy were also performed (Fig. 1, Extended Data Table 2 and Extended Data Fig. 2b). To further investigate the presence of HIV after allo-SCT, leukapheresis was performed 6.5 years after treatment interruption. Analysis of 109 million PBMCs identified potential traces of residual total HIV DNA, with a maximum frequency of 0.07 copies per million cells. Notably, at the same time point, no viral production was detected in a quantitative viral outgrowth assay using >130 million CD4 T cells, with an estimated lower limit of detection of less than 0.006 infectious units per million cells (Methods and Extended Data Table 2). Overall, these findings indicate a significant reduction and possibly complete eradication of replication-competent HIV after allo-SCT.

## CCR5 status and functionality

**Fig. 2:**
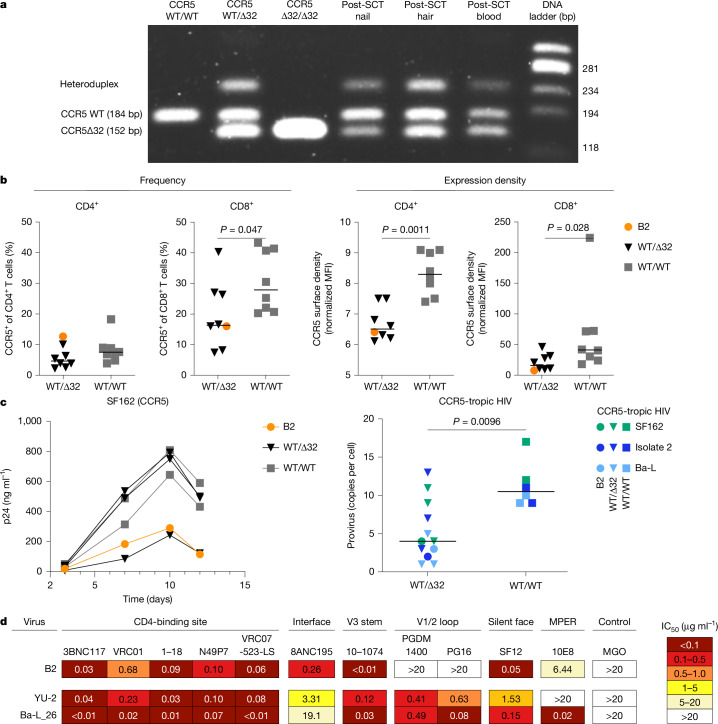
CCR5 status and susceptibility of PBMCs to HIV infection or autologous virus to broadly neutralizing antibody neutralization. **a**, Genotyping patterns of individuals with different CCR5 allele compositions depicted in the left three lanes (homozygous wild-type: WT/WT; heterozygous wild-type/Δ32: WT/Δ32 and homozygous Δ32: Δ32/Δ32). The right three lanes show the genotyping results for patient B2. Nail and hair samples correspond to the patient’s heterozygous WT/Δ32 genetic background. The post-SCT blood sample reflects the heterozygous WT/Δ32 genotype of the donor after transplantation and complete chimerism. The lane to the right shows the DNA ladder. Samples containing heterozygous WT/Δ32 alleles produce both bands, plus an additional third heteroduplex band arising from secondary structures of PCR products. Genotypic PCR was independently performed twice for all samples, with consistent results obtained. **b**, CCR5 phenotype determined as the percentage of CCR5-expressing cells and the expression density in the CD4 and CD8 T cell compartments, respectively. Triangles and squares depict individuals with heterozygous CCR5 WT/Δ32 or WT CCR5, respectively. Patient B2 is depicted by orange circles. *n* = 8 biologically independent samples per group. Significance was determined using two-tailed Mann–Whitney *U*-test. MFI, mean fluorescence intensity. **c**, PBMCs from patient B2 (orange circle) and individuals with heterozygous CCR5 WT/Δ32 (triangles) or WT/WT (as the control group; squares) were tested for susceptibility to HIV infection using CCR5-tropic HIV strains and primary isolates (CCR5: SF162, Ba-L and isolate 2). Productive HIV infection was detected by quantification of HIV-1 p24 protein and quantitative proviral PCR at day 12. Representative plots for SF162 infection and proviral PCR results after CCR5-tropic HIV challenge are shown on the left and right, respectively. *n* = 18 biologically independent samples. Significance was determined using two-tailed Mann–Whitney *U*-test. **d**, Neutralization sensitivity of autologous pseudovirus (B2) or standard HIV strains (YU-2 and Ba-L) was tested against a panel of broadly neutralizing antibodies (bNAbs) grouped by known epitope-binding regions on the HIV envelope protein. Neutralization titres are shown as half-maximal inhibitory concentration (IC_50_) values in μg ml^−1^. MPER, membrane proximal region.

Next, we confirmed that host-derived samples (fingernail and hair follicle) and post-allo-SCT blood samples had the heterozygous CCR5 wild-type/Δ32 genetic background. This result reflected the donor genetic background at complete chimerism (Fig. 2a). CCR5 haplotyping revealed that both donor and host share the HHC/HHG*2 haplotype pair, which was previously shown to be associated with favourable HIV clinical progression^23,24^ (Extended Data Fig. 1a).

**Extended Data Fig. 3:**
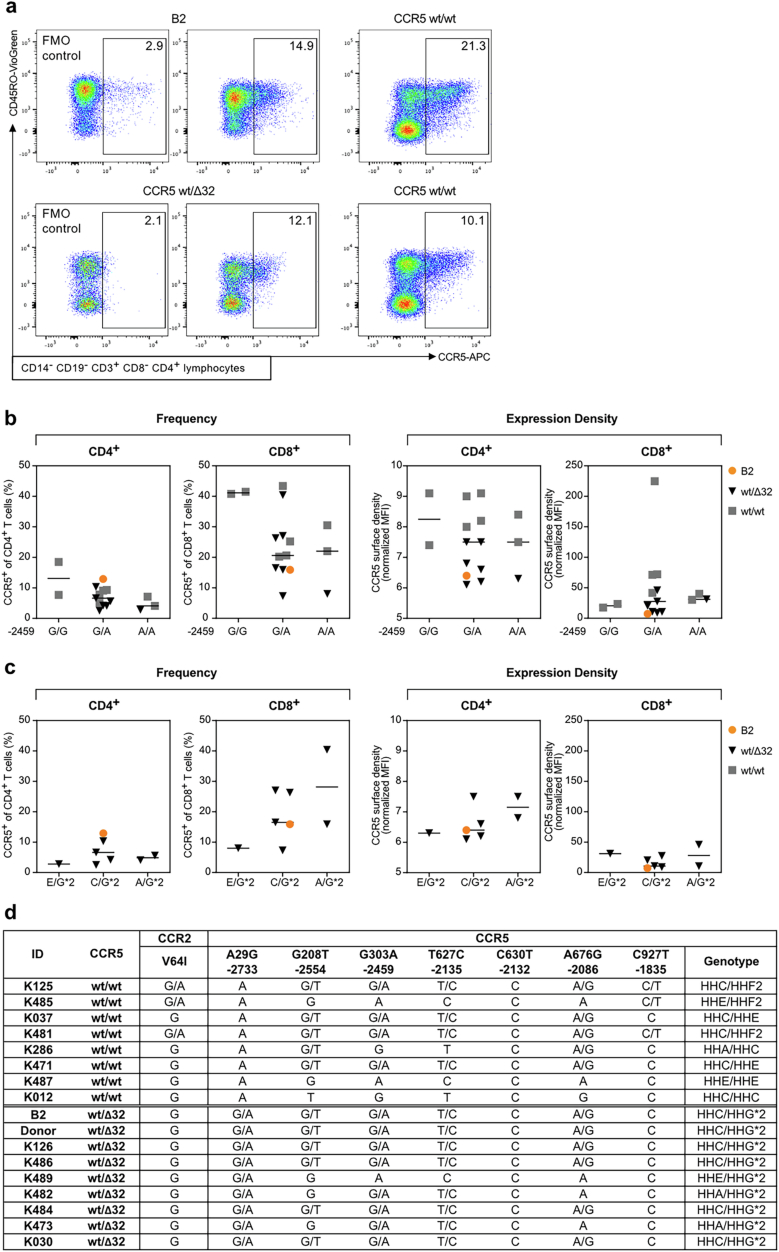
CCR5 phenotyping, polymorphisms and haplotypes. **a**, Representative flow cytometry plots from patient B2 and controls showing gating strategy for CCR5 expression. Background APC signal observed in patient B2 likely reflects cellular autofluorescence due to elevated activation of highly differentiated T cells. **b**, CCR5 frequency and expression density in the CD4 and CD8 T cell compartments of patient B2 and control individuals carrying the CCR5 −2459G/G, −2459G/A and −2459A/A polymorphism. Triangles and squares depict heterozygous CCR5 wild-type/Δ32 or wild-type CCR5 controls, respectively. Patient B2 depicted by orange circles. N = 16 independent biological samples. Statistical significance was determined using the Kruskal–Wallis test. **c**, CCR5 frequency and expression density in the CD4 and CD8 T cell compartments of patient B2 and heterozygous CCR5 wild-type/Δ32 control individuals, grouped by CCR5 haplotype pairs (E/G*2=HHE/HHG*2; C/G*2=HHC/HHG*2; A/G*2=HHA/HHG*2). Triangles depict heterozygous CCR5 wild-type/Δ32 controls. Patient B2 depicted by orange circles. N = 8 independent biological samples. Statistical significance was determined using the Kruskal–Wallis test. **d**, CCR5 haplotype analysis.

The relative frequency of CCR5-expressing CD4 T cells was comparable between individuals with wild-type CCR5 and individuals with heterozygous CCR5 wild-type/Δ32, including the B2 patient. However, CCR5 frequency in CD8 T cells and expression density in both CD4 and CD8 T cells were significantly lower in individuals with the heterozygous genotype than the wild-type (Fig. 2b and Extended Data Fig. 3a). No significant differences in CCR5 frequency or expression density were observed between patient B2 and individuals with specific CCR5 polymorphisms (position –2459G/G, G/A or A/A) or wild-type/Δ32 haplotypes (HHE/HHG*2, HHC/HHG*2 or HHA/HHG*2), who were used as the control group (Extended Data Fig. 3b–d).

**Extended Data Fig. 4:**
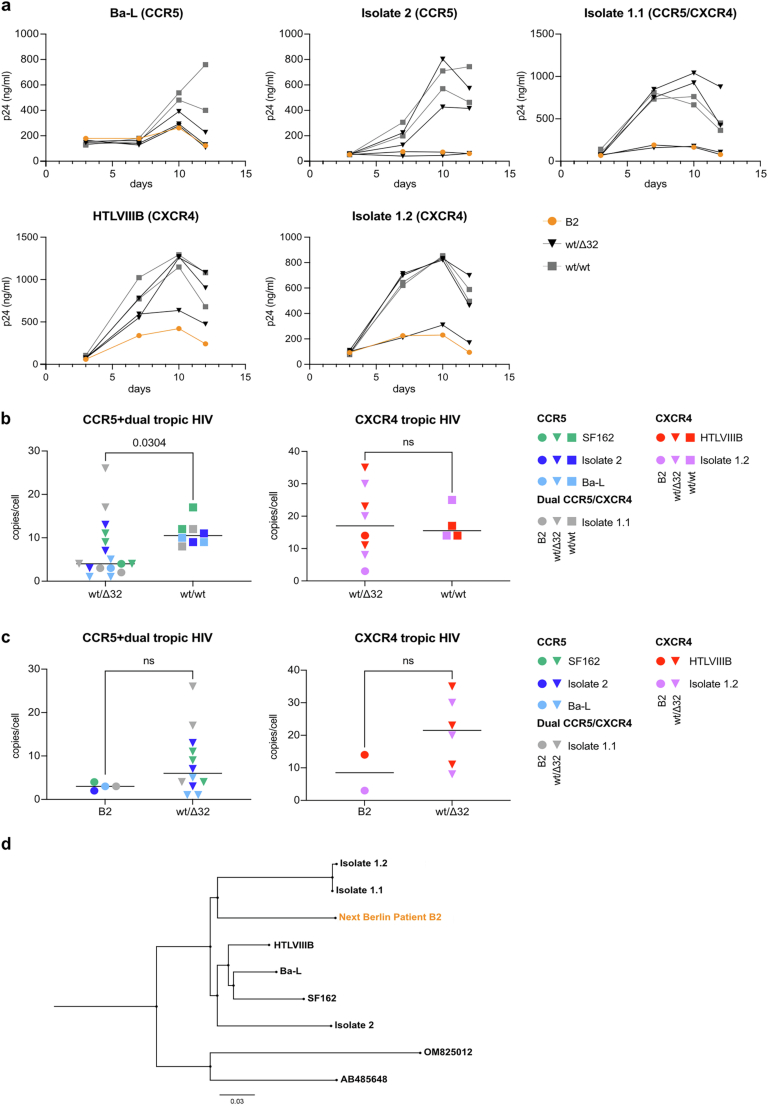
HIV susceptibility and phylogenetic tree. **a-c**, PBMCs from patient B2 (orange circle), CCR5 wt/Δ32 (triangles) or CCR5 wt/wt (squares) controls tested for susceptibility to HIV infection using CCR5-, CXCR4- or dual X4/R5-tropic HIV strains and primary isolates (CCR5: SF162 (green), Ba-L (light blue), Isolate 2 (blue); CXCR4: HTLVIIIB (red), Isolate1.2 (pink); dual X4/R5: Isolate1.1 (grey)). Productive HIV infection detected by quantification of HIV-1 p24 protein (a) and quantitative proviral PCR at day 12 (b and c). N = 24 (left) and n = 12 (right) biologically independent samples (b). N = 16 (left) and n = 8 (right) biologically independent samples (c). Statistical significance was determined using two-tailed Mann–Whitney U-test. **d**, Maximum likelihood phylogenetic tree displaying the patient env V1-V5 sequence (HXB2 nucleotides 6266–7765) recovered from plasma before allo-SCT. Two subtype D sequences (OM825012 and AB485648) were included as the outgroup in addition to reference (HTLVIIIB, Ba-L, and SF162) and German HIV-1 Seroconverter Study isolate sequences (Isolate 1.1, 1.2, and 2)^67^.

To test for HIV susceptibility, PBMCs from patient B2 and HIV-negative individuals with either heterozygous or wild-type CCR5 genotypes were challenged in vitro with CCR5-tropic, CXCR4-tropic or dual X4/R5-tropic HIV strains and primary isolates: SF162, Ba-L and isolate 2 for CCR5; HTLVIIIB and isolate 1.2 for CXCR4; and isolate 1.1 for dual X4/R5). Productive HIV infection was detected through the quantification of HIV-1 p24 protein and quantitative proviral PCR. PBMCs from patient B2 and the control group were generally susceptible to HIV infection, regardless of CCR5 status or the viral tropism of HIV strains and primary isolates (Fig. 2c and Extended Data Fig. 4a). Notably, levels of infection were significantly lower in individuals with the heterozygous genotype than in individuals with wild-type CCR5 after challenge with CCR5-tropic and CCR5^+^ dual X4/R5-tropic HIV (Fig. 2c and Extended Data Fig. 4b). By contrast, no difference was observed after CXCR4-tropic HIV infection (Extended Data Fig. 4b). Finally, although infection levels varied among individuals, there were no significant differences between patient B2 and the other individuals with heterozygous CCR5 wild-type/Δ32 in the control group after CCR5-tropic, CXCR4-tropic or dual X4/R5-tropic HIV infection (Extended Data Fig. 4c).

## Viral characteristics

To examine the characteristics of the patient-derived virus, we performed V3 loop sequencing and prediction of HIV-1 co-receptor usage by Geno2pheno computational algorithms^25^. Fewer than 0.3% of CXCR4-tropic viral sequences were detected, which confirmed the presence of CCR5 tropism of plasma autologous virus before ART initiation and allo-SCT (Methods). We also performed HIV-1 RNA extraction and single-genome amplification of the HIV-1 *env* gene from a pre-transplant plasma sample (Extended Data Fig. 4d). Recovered *env* was used to generate a pseudovirus with an autologous patient-derived envelope protein, which was tested for sensitivity to broadly neutralizing antibodies together with the standard HIV strains YU-2 and Ba-L for comparison (Fig. 2d and Methods). For patient B2-derived pseudovirus, we observed sensitivity to representative broadly neutralizing antibodies targeting the CD4 binding site and the V3 loop, with partial or complete resistance to broadly neutralizing antibodies directed against the membrane-proximal external region or the V1/2 loop of the HIV-1 envelope protein (Fig. 2d). Taken together, our findings confirm the infectivity, functional properties and susceptibility profiles of autologous virus from patient B2.

## HIV-specific immune responses

**Fig. 3:**
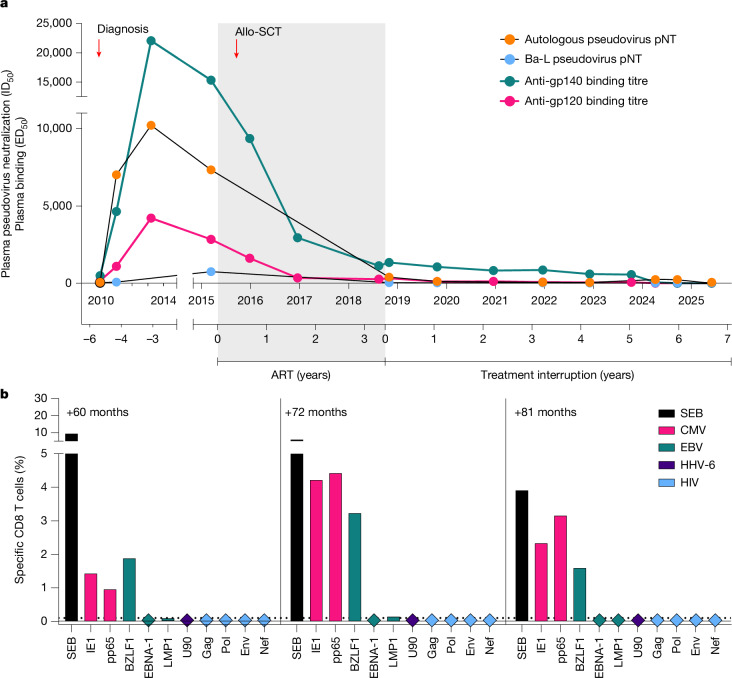
HIV-specific adaptive immune responses. **a**, Antibody response dynamics measured as plasma reactivity against HIV-1 Env proteins (gp120, pink; gp140, green) and pseudovirus-neutralizing activity (autologous pseudovirus, orange; Ba-L clade B pseudovirus, blue). pNT, pseudovirus neutralization titre. **b**, Virus-specific CD8 T cell responses (measured as production of interferon-γ (IFNγ), tumour necrosis factor (TNF) or interleukin-2 (IL-2)) against CMV (pink), EBV (green), HHV-6 (purple) and HIV (light blue) peptide pools at 60, 72 and 81 months after ART interruption. *Staphylococcus* enterotoxin B (SEB, black) represents the positive control.

**Extended Data Fig. 5:**
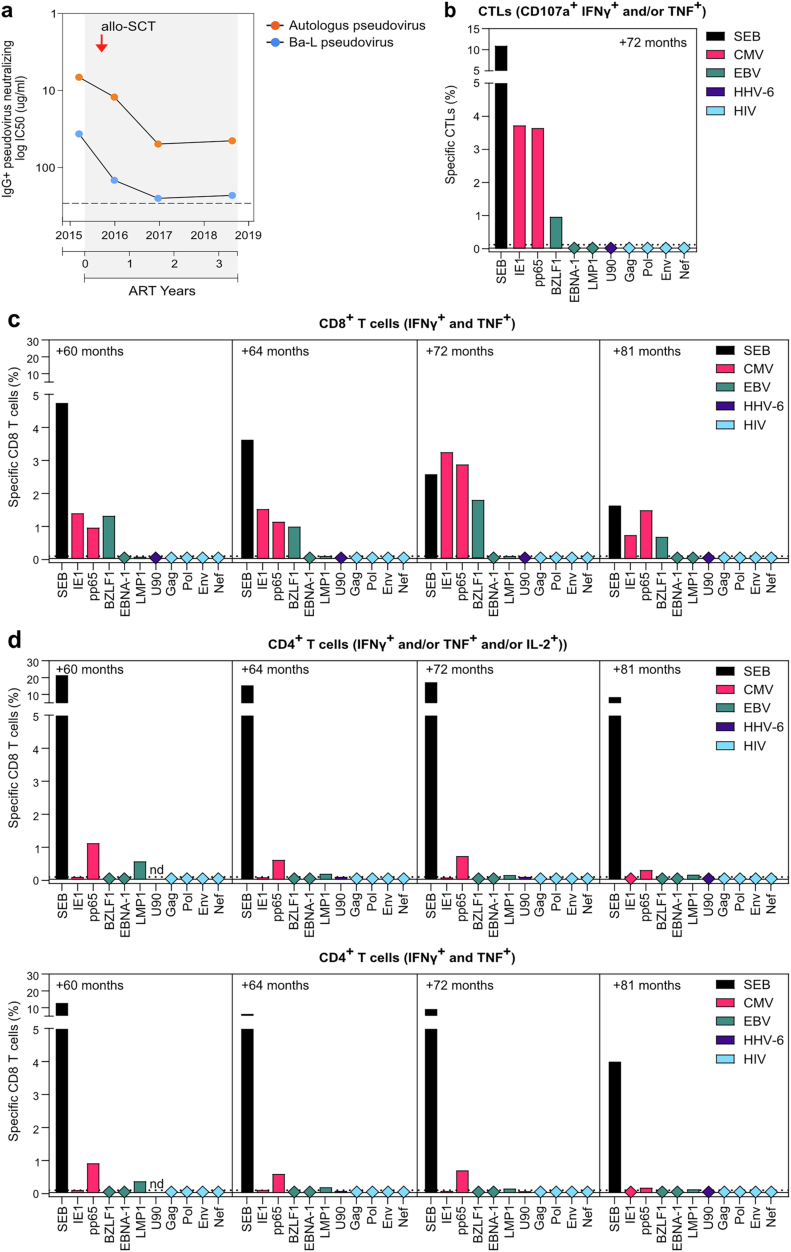
IgG neutralization, CD8 degranulation, CD4 T Cell stimulation assays. **a**, Antibody response dynamics measured as plasma purified IgG antibodies pseudovirus neutralizing activity (autologous pseudovirus, orange; Ba-L clade B pseudovirus, blue). **b-d**, Virus-specific cytotoxic T cell responses (measured as CD107a externalization together with IFNγ and/or TNF expression) against CMV (pink), EBV (green), HHV-6 (purple) and HIV (light blue) peptide pools at 72 months after ART interruption. Virus-specific CD8 (c) or CD4 (d) T cells responses (measured as production of IFNγ, TNF and IL-2) against CMV (pink), EBV (green), HHV-6 (purple) and HIV (light blue) peptide pools at 60, 64, 72 and 81 months after ART interruption. Staphylococcus enterotoxin B (SEB, black) represents positive control.

**Extended Data Fig. 6:**
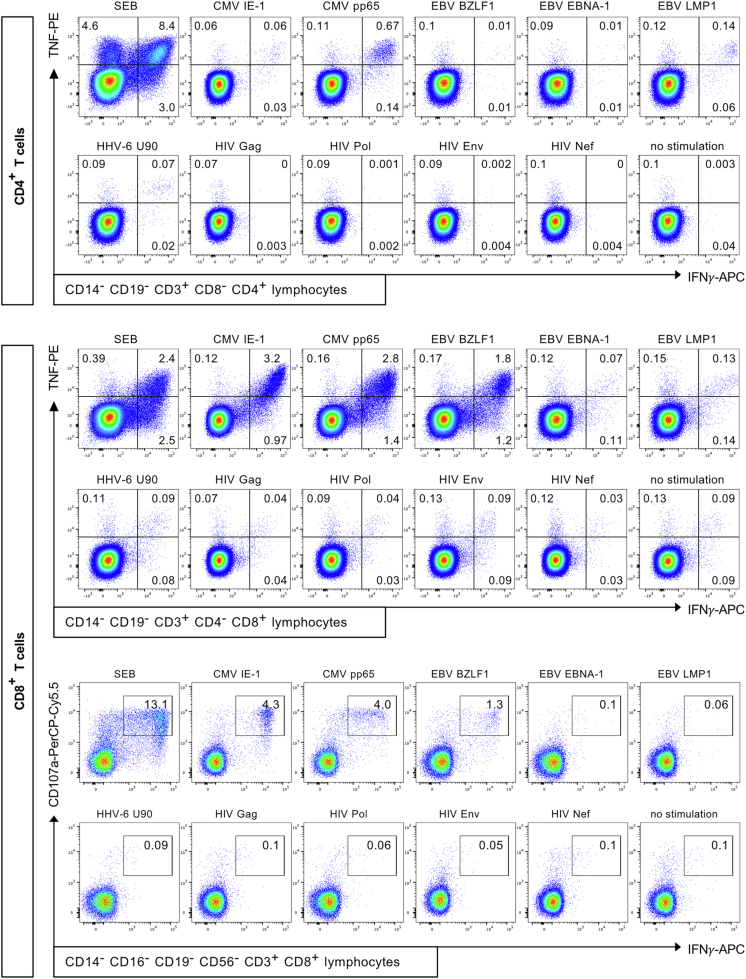
T cell stimulation assays. Representative flow cytometry plots from CD4 and CD8 T cell stimulation assays.

To further assess potential loss of persistent viral infection or antigenic stimulation, we analysed HIV-specific antibody and T cell responses over time. IgG antibodies against the envelope proteins gp140 and gp120 detected by ELISA increased after HIV diagnosis. By contrast, HIV-specific antibody levels gradually decreased to low or undetectable after allo-SCT (Fig. 3a). Moreover, although potent neutralization of autologous and Ba-L.26 clade B pseudovirus was observed in plasma and purified IgG before allo-SCT, neutralizing activity declined to minimal residual levels in the years after transplantation (Fig. 3a and Extended Data Fig. 5a). HIV-specific T cell responses have recently been proposed as a sensitive surrogate marker for viral persistence or re-exposure in transplant recipients with undetectable reservoirs^7^. To determine HIV-specific T cell responses, we performed intracellular cytokine staining and degranulation assays after stimulation with HIV-1 Gag, Pol, Nef and Env or control peptide pools from human cytomegalovirus (CMV), Epstein–Barr virus (EBV) and human herpesvirus type-6 (HHV-6). No HIV-1-specific CD4 or CD8 T cells were detected in repeated measurements after allo-SCT. By contrast, persistent CMV-specific, EBV-specific and HHV-6-specific responses were detected in the CD4 and CD8 T cell compartments (Fig. 3b and Extended Data Figs. 5b–d and 6). Overall, these findings indicate the lack of antigenic stimulation or immune-mediated control sustaining HIV remission in the absence of ART after 6 years of treatment interruption.

## Natural killer cell immunity

**Fig. 4:**
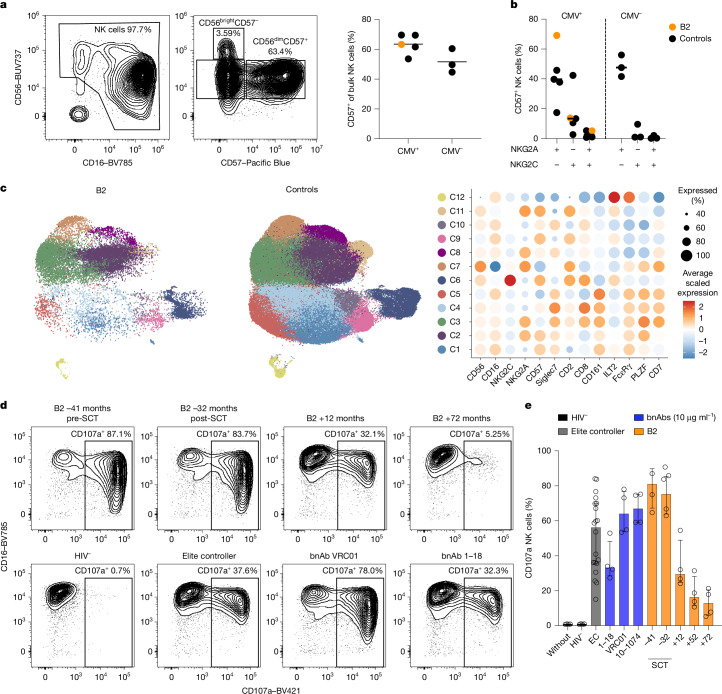
NK cell immunity. **a**, Phenotypic distribution of NK cell subsets of patient B2 using CD56 and CD16 (left) and subsequent gating on CD56 and CD57 (middle). The scatter plot (right) depicts the frequency of CD57 expression on NK cells in CMV^+^ and CMV^−^ individuals (as the control group). Data are shown for *N* = 7 biologically independent HIV^−^ donors (black, CMV^+^, *N* = 4 and CMV^−^, *N* = 3) and B2 (orange). No statistical tests were performed. Horizontal lines represent the median for each group. **b**, Scatter plot depicting frequencies of NKG2A and NKG2C NK cell subset combinations on CD57^+^ NK cells in CMV^+^ and CMV^−^ donors. Data are shown for *N* = 7 biologically independent HIV^−^ donors (black, CMV^+^, *N* = 4 and CMV^−^, *N* = 3) and B2 (orange). No statistical tests were performed. Horizontal lines represent the median for each group. **c**, Uniform manifold approximation and projection (UMAP) plot of bulk NK cells from patient B2 (left) with a corresponding UMAP plot from patient B2 and individuals in the control group (*N* = 4 CMV^+^, *N* = 3 CMV^–^). Samples are coloured based on 12 clusters, which were obtained by merging initial Louvain clusters that shared similar identities. The dot plot on the right shows the average scaled expression of selected NK cell markers for each cluster (C1–C12). **d**, Representative contour plots showing CD107a surface expression on NK cells after the ADCC assay. NK cells were incubated with Env-coated wells pretreated with longitudinal plasma samples from B2 (−41 pre-SCT, −32, +12 and +72 months; top row from left to right), plasma from a HIV^–^ donor, an elite controller or the bNAbs VRC01 and 1–18 (bottom row from left to right). **e**, Summary of cumulative ADCC responses. NK cells (*n* = 4 biological replicates) were incubated with Env-coated wells treated with PBS, plasma from a HIV^–^ donor (black), plasma from five elite controllers (EC, grey), bNAbs (1–18, VRC01 and 10–1074; blue) or longitudinal B2 plasma samples (orange). Bars indicate median with interquartile range.

**Extended Data Fig. 7:**
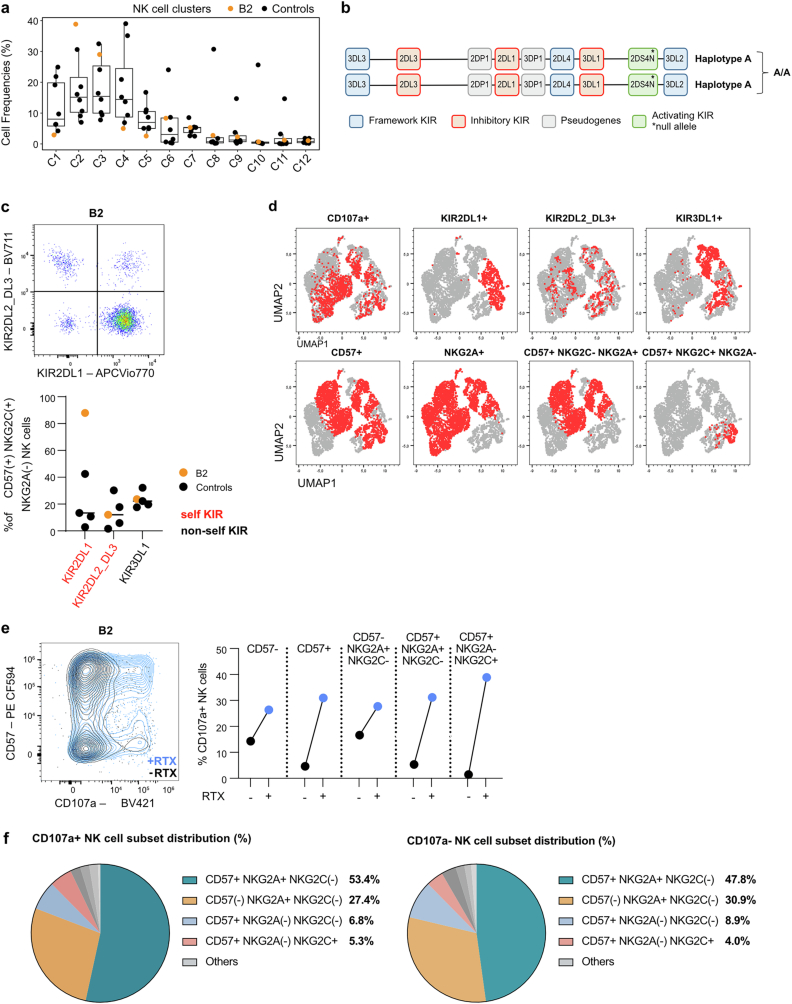
NK cells. **a**, Boxplot showing the input normalized frequency of cells per cluster for each donor. Data are shown for n = 7 biologically independent HIV− donors (black circles, CMV+: n = 4 and CMV−: n = 3) in addition to B2 (orange). No statistical tests were performed. The line inside each box shows the median. Each box spans from the first to the third quartile. The Whiskers extend to the most extreme values within 1.5 × inter-quartile range from the lower and upper hinges. **b**, Schematic representation of the predicted KIR genotype present in patient B2. **c**, Flow cytometric dot plot (left) from patient B2 depicting self-KIR expression in NKG2A− NKG2C+ CD57+ NK cells. Scatter plots showing the frequency of self-KIR KIR2DL1, KIR2DL2_DL3, and non-self KIR KIR3DL1 in NKG2A− NKG2C+ CD57+ NK cells. Data are shown for n = 4 biologically independent HIV− and CMV+ donors (black circles) in addition to B2 (orange). No statistical tests were performed. **d**, UMAP plot of NK cells from B2 co-incubated with rituximab-coated Raji cells, with the indicated subset (above) highlighted in red. **e**, Contour plot showing CD107a expression in NK cells after co-culture with Raji cells (black) and rituximab-coated (RTX) Raji cells (blue). The scatter plot (middle) shows % of CD107a positive cells in gated NK cell subsets (indicated above) after co-culture with Raji cells (black) and RTX Raji cells (blue). Pie chart on the right illustrates the relative frequencies and distribution of CD107a positive NK cell subsets from B2 after co-incubation with rituximab-coated Raji cells, categorized by their expression of NKG2A, NKG2C, and CD57, with their relative frequencies displayed as percentages. **f**, Pie charts illustrates the relative frequencies and distribution of CD107a-positive (left panel) and -negative (right panel) NK cell subsets from B2 after co-incubation with rituximab-coated Raji cells, categorized by their expression of NKG2A, NKG2C, and CD57, with their relative frequencies displayed as percentages.

To examine innate immune responses and the potential contribution of antibody-dependent cellular cytotoxicity (ADCC) to reservoir clearance, we analysed the phenotype, genotype and functional capacity of natural killer (NK) cells in patient B2 after allo-SCT. We observed a regular distribution of NK cell subsets in peripheral blood, with CD56^dim^CD57^+^ NK cell frequencies similar to individuals who were HIV-1-negative and CMV-positive (Fig. 4a). No preferential expansion of NKG2A^+^NKG2C^+^CD57^+^ NK cells, a subset recently linked to elite control of HIV infection^26^, was detected in patient B2. By contrast, the proportion of NKG2A^+^NKG2C^–^CD57^+^ NK cells was unusually high in patient B2 compared with the individuals in our control group and previous reports including allo-SCT recipients^27,28^ (Fig. 4b). Unsupervised clustering analysis of flow cytometry data identified an adaptive NK cell cluster (C6) in individuals who were CMV-positive and in patient B2, as well as enrichment of cluster C2 in patient B2, which corresponded to the expanded NKG2A^+^CD57^+^ NK cell subset that lacks adaptive NK cell features such as reduced levels of PLZF and FcɛRγ (Fig. 4c and Extended Data Fig. 7a).

Killer cell immunoglobulin-like receptor (KIR) and HLA class I genotyping revealed the absence of distinct KIR–HLA combinations that were previously associated with HIV-1 immune control in untreated individuals (for example, *KIR3DS1* and *KIR3DL1–HLA-B Bw4*)^29–31^ or in ‘post-treatment controllers’^32^ living with HIV (Extended Data Fig. 7b). Moreover, high expression of the inhibitory KIR2DL1 on the adaptive cluster C6 is consistent with previous reports on CMV-positive blood donors^33^ (Extended Data Fig. 7c).

Next, we tested for NK cell function and ADCC activity in standardized degranulation assays. Adaptive NKG2C^+^CD57^+^ NK cells from patient B2 showed strong degranulation in response to antibody-coated targets. Moreover, owing to its high frequency, the NKG2A^+^CD57^+^ subset contributed substantially to the degranulating NK cell population (Extended Data Fig. 7d–f and Supplementary Fig. 1).

**Extended Data Fig. 8:**
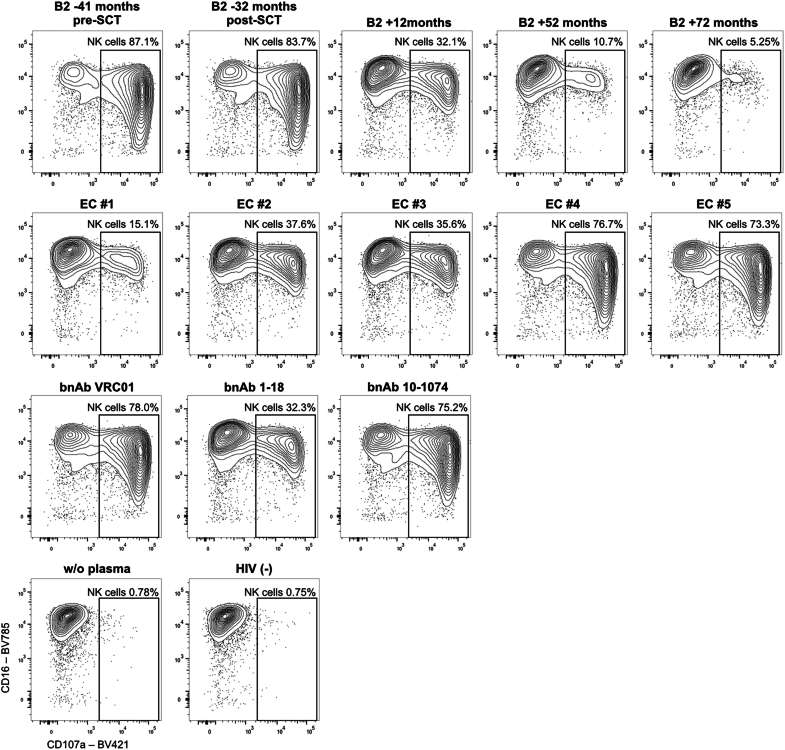
CD107a surface expression on NK cells following the ADCC assay. Contour plots showing CD107a surface expression on NK cells following the ADCC assay using immobilized HIV-1 Ba-L Env protein. NK cells were incubated with Env-coated wells pre-treated with longitudinal plasma samples from B2 (− 41 months (pre-SCT), −32 months (post-SCT), +12 months, +52 months and +72 months before or after ART interruption, respectively; top row from left to right), plasma from five elite controllers (second row), the bnAbs VRC01, 1-18 and 10-1074 (bottom row, first three plots from left to right) and without plasma or an HIV (-) donor (bottom row, last two plots from left to right). ADCC activity was assessed by CD107a surface expression after 4 h.

Given the potent HIV-specific antibody response around the time of transplantation, we next evaluated HIV-specific ADCC activity in the plasma of patient B2 over time. We assayed NK cell degranulation from HIV-negative donors following exposure to HIV-env antigens (Ba-L) in the presence of B2 plasma (pre-SCT and post-SCT), elite controller plasma (*n* = 5) or monoclonal broadly neutralizing antibodies (10–1074, VRC01 and 1–18). Notably, we detected the highest ADCC activity in plasma from patient B2 around the time of transplantation (degranulation median pre-SCT = 79.3%, post-SCT = 74.8%), exceeding ADCC responses induced by elite controller plasma (degranulation median = 51.9%) and monoclonal broadly neutralizing antibodies at 10 µg ml^–1^ (degranulation median ranging from 36.6% to 67%) (Fig. 4d,e and Extended Data Fig. 8). In parallel to antibody binding and neutralization, ADCC activity mediated by plasma from patient B2 declined significantly in the years after transplantation. Overall, we observed an NK cell phenotype with marked expansion of ADCC-responsive NKG2A^+^CD57^+^ NK cells, along with highly potent HIV-specific ADCC plasma activity in patient B2 around the time of allo-SCT, which may have contributed to clearance of the HIV reservoir.

## Mathematical modelling of viral rebound

**Fig. 5:**
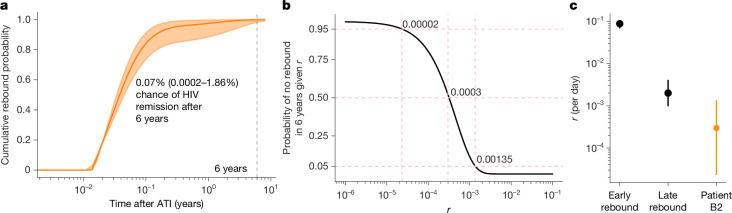
Modelling rebound after treatment interruption. **a**, Cumulative rebound probability from a data-validated model from ATI cohorts (*N* = 235, Methods) inclusive of late rebound with a decreasing recrudescence rate over time. The *x* axis is log_10_ scale. The line represents output using mean parameter value estimates and the shaded area covers the range outputted by using 5% and 95% parameter value estimates. The probability of HIV remission by 6 years is indicated by a vertical dashed line. **b**, Probability of no rebound in 6 years based on a model with an unknown recrudescence rate. The graph depicts the recrudescence rate for patient B2 as 5%, 50% and 95% chance. **c**, Values of early and long-term recrudescence rate from ATI cohorts versus an analytically calculated estimate for patient B2. Circles depict the mean, vertical lines represent 95% CI on recrudescence rates from the analytical calculations.

To determine the likelihood of observed HIV remission and the probability of viral reactivation, we applied a mathematical model of rebound time that was trained on data from large analytical treatment interruption (ATI) trials^34,35^. Unlike previous models^36,37^, this approach accounted for early-occurring and late-occurring viral rebound (less or greater than 60 days after ATI, respectively) by allowing the per day rate of viral rebound (recrudescence rate, *r*) to decrease with time after treatment interruption. Using this model, we estimated an extremely low probability (0.07%, confidence interval (CI): 0.0002–1.86%) of maintaining viral suppression without rebound for 6 years in the absence of ART (Fig. 5a).

Next, we used a more traditional rebound model to estimate the probability for viral reactivation within the 6-year timeframe based on the recrudescence rate (mean, with 5% and 95% CIs) for patient B2 (Fig. 5b). We estimated *r* to range between 0.00135 and 0.00002 per day, corresponding to a 95% probability of viral rebound occurring within 2–137 years, assuming that no rebound has occurred so far. The mean recrudescence rate for patient B2 was 300-fold and 7-fold lower than the mean rates from individuals who experienced early or late viral rebound, respectively, in ATI studies (Fig. 5c). Overall, these modelling results suggest that although not zero, the probability of viral rebound in patient B2 is exceedingly low (Fig. 5c).

## Discussion

The case of patient B2 demonstrates that durable HIV remission and potential cure can be achieved with functional viral co-receptors after heterozygous CCR5 wild-type/Δ32 allo-SCT. Notably, except for the individuals known as the first Berlin patient^1^ and the London patient^2^, patient B2 attained longer HIV remission than previous cure cases^3–6^, which underscores that homozygous CCR5Δ32-mediated viral resistance is not essential for achieving multiyear long HIV remission. These results suggest that the pool of stem cell donors with potential HIV cure implications could be expanded to include heterozygous CCR5Δ32 individuals, thereby overcoming the scarcity of homozygous CCR5Δ32 donors for patients with HIV requiring allo-SCT^38^.

Although the precise mechanisms that enable HIV cure despite non-resistant transplants remain uncertain, growing evidence from clinical cohorts and animal models highlights the role of allogeneic immunity in clearing persistent HIV with beneficial graft-versus-reservoir (GVR) effects mediating significant reductions in HIV-infected cells after allo-SCT^7,8^. In the case of patient B2, donor–recipient HLA-DPB1 mismatches and the presence of multiple miHA disparities predicted to be highly expressed in CD4 T cells may have contributed to beneficial GVR effects and HIV clearance. However, functional analyses are needed to determine the specific impact of miHA-directed T cell responses on outcomes.

In line with these findings, HIV reservoir analyses in patient B2 revealed a high percentage of genetically intact proviral HIV before transplantation, but no detectable replication-competent virus in blood or intestinal tissues after allo-SCT. Although the pre-SCT measurement was obtained during viraemia rather than under full viral suppression, recent studies indicate that reservoir levels remain relatively stable after ART initiation during chronic infection, which suggests that our baseline provides a reasonable qualitative and quantitative approximation of the reservoir under suppression^39,40^. Thus, although persistence of intact HIV in host-derived CD4 T cells cannot be completely ruled out, the absence of viral activity for over 6 years suggests a substantial reduction, or possibly complete eradication, of the biologically relevant HIV reservoir in patient B2. Furthermore, declining or absent HIV-specific antibody and cellular immune responses indicate that no viral antigens were produced after treatment interruption. Notably, patient B2 did not require prolonged immunosuppression for GVHD, a treatment that might have contributed to preventing viral reactivation in previous cure cases^3,5,6,41^.

Immune memory is not limited to adaptive T and B cells, as NK cells can also demonstrate long-lasting, memory-like responses^42^. Moreover, certain NK cell subsets and receptor–ligand combinations have been linked to HIV immune control^26,29^ and enhanced reservoir clearance through ADCC^43,44^. In patient B2, potent plasma ADCC activity around the time of transplantation, together with an expanded NKG2A^+^CD57^+^ NK cell subset, may have facilitated HIV reservoir clearance by eliminating infected cells^45,46^.

Finally, we propose that the underlying heterozygous CCR5Δ32 status of patient B2 may have significantly contributed to achieving durable HIV remission. Patient B2 showed a favourable clinical course before treatment initiation, which may be linked to the CCR5 HHC/HHG*2 haplotype^23,24^. Notably, three out of the seven individuals reportedly cured of HIV, including patient B2, were heterozygous for CCR5Δ32 (refs. ^1,3^). Moreover, two individuals known as the Boston patients who achieved prolonged viral suppression despite wild-type CCR5 transplants were also heterozygous for CCR5Δ32 (refs. ^12,47^). CCR5Δ32 heterozygosity has been associated with lower viral load set points, slower disease progression and may affect HIV evolutionary dynamics by limited CCR5 availability^16,48,49^. Low CCR5 expression has been linked to natural HIV control^50^, and recently, higher prevalence of CCR5Δ32 heterozygosity has been observed among viraemic non-progressors, a rare group of people living with HIV who preserve immune function despite uncontrolled viral replication^51^. Furthermore, in a CCR5-targeting gene therapy study, individuals with heterozygous CCR5Δ32 were more likely to achieve viral control^52^.

In patient B2 and other individuals with heterozygous CCR5 (as the control group), we observed significantly lower CCR5 expression and infection susceptibility compared with individuals with wild-type CCR5. Although a large cohort study detected no differences in reservoir size between individuals with heterozygous CCR5 and those with wild-type CCR5, as measured by proviral HIV-1 DNA and cell-associated RNA in peripheral blood^53^, other studies reported favourable effects of monoallelic CCR5 expression on the size and formation of viral reservoirs^51,54–56^. Although the exact mechanisms remain to be elucidated, we speculate that CCR5Δ32 heterozygosity may influence the formation, composition and tissue distribution of HIV reservoirs, thereby potentially leading to more efficient elimination after allo-SCT. Of note, patient B2 achieved viral suppression shortly before allo-SCT, similar to the first Berlin patient, Timothy Brown, who experienced viraemia before his first transplant following a period of treatment interruption^1^. Further investigation and detailed characterization of HIV reservoir biology are needed to determine the impact of treatment initiation before transplantation and the potential beneficial effects of monoallelic CCR5 expression.

Overall, the case of the second Berlin patient B2 suggests that significant reductions in persistent reservoirs can lead to HIV cure independent of homozygous CCR5Δ32-mediated viral resistance. This finding underscores the critical importance of modulating and potentially eliminating the HIV reservoir in strategies aimed at long-term remission and cure.

## Methods

### Ethics

Written informed consent was obtained from patient B2 following consultation with the local Ethics Committee of Charité Universitätsmedizin Berlin. Biological samples from patient B2 were used for research purposes in accordance with the Ethics Committee of Charité Universitätsmedizin Berlin (reference number EA4/261/23). The decision to discontinue ART was made independently by patient B2. Participants in the control group, including HIV-negative blood donors and individuals living with HIV, were enrolled at Charité Universitätsmedizin Berlin and the University Medical Center Hamburg-Eppendorf under approved ethical protocols (Ethics Committee of Charité Universitätsmedizin Berlin, reference numbers EA2/077/23 and EA4/255/23; and Ärztekammer Hamburg, reference number PV4780). Biological samples from patients who received allo-SCTs without HIV infection were previously collected as part of a previous study^57^. Written informed consent was obtained from all participants, and the studies were conducted in accordance with Good Clinical Practice.

### Sample processing

Blood samples were collected and processed according to previously established protocols^58^. Serum and plasma samples were stored at −80 °C. PBMCs were isolated by density gradient centrifugation (1.077 g ml^–1^ Pancoll, PAN Biotech) and cryopreserved in fetal bovine serum plus 10% DMSO before storage in liquid nitrogen.

### Detection and quantification of HIV-1 RNA plasma viral load

Plasma viral load was measured until December 2019 with an Abbott RealTime HIV-1 assay (2G31-090) using m2000sp automated nucleic acid extraction and a m2000rt realtime-PCR cycler, which has a lower limit of detection (LLOD) of 25 copies per ml. From December 2019 onwards, we used an Abbott Alinity m HIV-1 AMP Kit (8N45-090) on the Alinity m platform with a LLOD of 14.0 copies per ml. After September 2023, testing and quantification for HIV-1 RNA was done using a Cobas HIV-1 assay (Roche). The analytical sensitivity (LOD by hit rate of ≥95%) of this assay is 14.2 copies per ml and the linear range for quantification is 20−1 × 10^7^ copies per ml.

### Chimerism

Chimerism analyses were based on the discrimination of donor and recipient alleles on short tandem repeats (STRs) using PCR with fluorescence-labelled primers and DNA fragment analysis. Initial genotyping to detect informative STR loci was performed using EDTA peripheral blood from the patient and donor or graft. After transplantation, chimerism was analysed in bone marrow and peripheral blood samples. Isolation of CD34^+^ (from bone marrow) and CD3^+^ (from peripheral blood) cells was performed using a standard MACS technique (Miltenyi Biotec). DNA was extracted using a standard DNA extraction kit (QIAamp; Qiagen), as recommended by the manufacturer. The PCR reaction was run using a commercial AmpFℓSTR Identifier PCR Amplification Kit (Thermo Fisher Scientific), which contains fluorescent-labelled primer pairs for simultaneous amplification of 16 different loci. For quantification of chimerism, the areas under the curves were calculated using GeneMapper (v.3.7) software (Thermo Fisher Scientific). The sensitivity of the method is 1%.

### Whole-exome sequencing

Genomic germline DNA from the donor and the recipient were quantified using a Qubit Fluorometer, and DNA integrity was assessed using a TapeStation (Agilent). A total of 200 ng of input genomic DNA (gDNA) was enzymatically fragmented for 25 min using an Agilent SureSelect Enzymatic Fragmentation Kit (Agilent). Library preparation was performed using an Agilent SureSelect XT HS2 DNA Reagent Kit with AMPure XP/Streptavidin Beads (Agilent) and Agilent Human All Exon v8 enrichment baits (5191-6875, Agilent) according to the manufacturer’s recommendations. Libraries were pooled to equimolar concentrations based on Qubit concentration measurements and TapeStation size distributions. The loading concentration of the pool was determined using a qPCR assay (Roche). Libraries were sequenced on an Illumina NovaSeq X Plus platform using PE100 sequencing mode, with a target of 50 million reads per sample (around 75× coverage).

### Prediction of candidate miHAs

The pipeline for systematic miHA discovery from donor and recipient whole-exome sequencing data was adopted from a previous study^18^, and sequencing reads were aligned to the hg38 reference genome with BWA. Germline variant calling and functional SNP annotation were performed using Deepvariant^59^ and Funcotator (GATK4), respectively. Exonic non-synonymous variants from the donor and the recipient were compared, and the discordant variants present only in the recipient were identified. The GVL filter has been previously published^18^ and is composed of the AML filter and the haematopoietic filter, which contain 650 genes. The CD4 T cell filter was generated on the basis of 7,208 genes expressed in more than 5% of CD4 T cells in healthy donor PBMC datasets (https://atlas.fredhutch.org/nygc/multimodal-pbmc/)^60^. The discordant variants present in genes from the GVL and CD4 T cell filters were selected, and all possible *k*-mers encompassing the variants were generated. Moreover, possible *k*-mers arising from genes in the male-specific Y chromosome region (nine genes) were generated as additional targets of allo-recognition in female-to-male transplant. All *k*-mers were sequentially blasted against custom in silico proteomes inferred from the exomes of both the donor and the recipient, and all *k*-mers found in unrelated tissue sites were discarded. The remaining unique *k*-mers were subjected to HLAthena^61^ to predict the binding affinity with patient-specific HLA class I alleles using a threshold of 0.5% prediction rank to define binders.

### Antiretroviral drug screening in plasma

Plasma levels of HIV drugs were measured using an in-house method based on reverse-phase LC–MS/MS in multiple-reaction monitoring mode to check for the presence of amprenavir, darunavir, efavirenz, indinavir, lopinavir, maraviroc, nelfinavir, nevirapin, raltegravir, ritonavir, saquinavir, etravirin, elvitegravir, dolutegravir, tipranavir, tenofovir and emtricitabine, with a LLOD of 25 ng ml^–1^ plasma.

### Mucosal cell isolation

Mucosal mononuclear cells (MMCs) were isolated from 16 duodenal or 20 ileal biopsies through treatment with 1 mM EDTA and digestion with collagenase type II (Sigma) followed by Percoll gradient centrifugation^62^. Antibody clones used in flow cytometry panels were tested to be suitable for the analysis of collagenase-treated cells. For phenotyping, the following antibodies were used: anti-CD3-PerCP (SK7; BD, dilution 1:20); anti-CD4-Pacific Blue (RPA-T4; BD, dilution 1:25); anti-CD19-PE-Cy7 (SJ25C1; BD, dilution 1:50); anti-CD45-FITC (HI30; eBioscience, dilution 1:25); anti-CD326-PE-Cy7 (9C4; BioLegend, dilution 1:50); and anti-HLA-DR-PE (LN3; eBioscience, dilution 1:10). CD45^+^CD4^+^ cells were purified from MMCs with the use of anti-CD4-FITC (RPA-T4; BD, dilution 1:20), anti-CD45-V500 (HI30; BD, dilution 1:50) and anti-CD326-PE-Cy7 (dilution 1:50) by flow sorting using a FACSJazz cytometer (BD).

### Total HIV DNA

For detection of total proviral HIV-1 DNA, gDNA was isolated from peripheral blood using a QIAamp DNA Blood Mini Kit (Qiagen) or a Maxwell RSC Whole Blood DNA Kit (Promega). PCR involved two independent reactions targeting the *env* and long-terminal-repeat regions as previously described^1^.

### Total HIV-1 DNA in gastrointestinal biopsy samples and PBMCs

Proviral HIV DNA was assessed in mononuclear cells isolated from the lamina propria of the duodenum or ileum and in CD45^+^ CD4 cells from the duodenum by PCR according to previously established protocols^1,57^. For additional reservoir quantification by digital PCR (dPCR), four aliquots of MMCs were resuspended in 200 µl PBS and DNA was isolated using a QIAmp DNA Mini Kit (Qiagen). To quantify the genomic yield, dPCR using the RPP30 assay^22^ was performed on 20 ng for the PBMC eluate and 5 µl of each gastrointestinal biopsy eluate. For the detection of potential HIV reservoirs, 500 ng gDNA from PBMCs or 20 µl from four aliquots from the gastrointestinal biopsy was tested for total HIV DNA (RU5)^63^ by dPCR in double testing. dPCR reservoir measurements from intestinal biopsy samples and PBMC eluate were performed on a QIAcuity One 5-plex (Qiagen).

### Triplex IPDA

CD4 cells were isolated from 20 ml peripheral blood by positive selection (Dynabeads CD4, Thermo Fisher Scientific). CD4 cell DNA was isolated in three aliquots. Shear factor and genomic yield were assessed using the RPP30 assay^22^ in 20 ng DNA templates. The Triplex IPDA includes total HIV DNA (RU5)^63^ and the quantification of intact proviral HIV DNA (packaging signal (PSI), Env and an unlabelled Env hypermutated probe)^21^ in 500 ng templates per well with at least triple testing. All dPCR assays were performed on a QIAcuity One 5-plex (Qiagen).

### Q4ddPCR

CD4 T cells were isolated from samples using a CD4 T Cell Isolation Kit (Miltenyi Biotec) according to the manufacturer’s instructions. gDNA was then extracted using a QIAamp DNA Mini Kit (Qiagen). Up to 750 ng of DNA per well was combined with supermix for probes (no dUTP) (Bio-Rad) and a custom primer–probe mix. This mix included four fluorescently labelled internal hydrolysis probes along with an unlabelled hypermutant Env probe at varying final concentrations. The primer and probe sequences were based on published sequences, with modifications to fluorophore labelling^21,64^. The sequences for each target are as follows: Env: primer (0.225 µM): forward AGTGGTGCAGAGAGAAAAAAGAGC, reverse GTCTGGCCTGTACCGTCAGC, probe (0.0625 µM): /5-VIC-CCTTGGGTTCTTGGGA-MGBNFQ, unlabelled hypermutant probe for discrimination of hypermutations in the target: CCTTAGGTTCTTAGGAGC-MGBNFQ; PSI: primer (0.9 µM): forward CAGGACTCGGCTTGCTGAAG, reverse GCACCCATCTCTCTCCTTCTAGC, probe (0.25 µM): /56-FAM/TTTTGGCGTACTCACCAGT-MGBNFQ); Gag: primer (0.9 µM): forward ATG TTT TCA GCA TTA TCA GAA GGA, reverse TGC TTG ATG TCC CCC CAC T, probe (0.25 µM): /5Cy5/CCACCCCAC/TAO/AAGATTTAAACACCATGCTAA/3IAbRQSp/); Pol: primer (0.9 µM): forward GCA CTT TAA ATT TTC CCA TTA GTC CTA, reverse CAA ATT TCT ACT AAT GCT TTT ATT TTT TC, probe (0.25 µM): /5ATTO590N/AAGCCAGGAATGGATGGCC/3IAbRQSp/). Primers and probes were purchased from Integrated DNA Technologies, except for the Env and PSI probes, which contained a minor groove binder (Thermo Fisher Scientific). Reactions were performed in a total volume of 20 µl per well in up to four replicate wells. Droplets were generated with an automated droplet generator (Bio-Rad). Thermocycling was performed with a 2 °C ramp rate, with an initial denaturation at 95 °C for 10 min, followed by 60 cycles (30 s at 94 °C and 1 min at 55 °C per cycle) and a final step 10 min at 98 °C before incubation at 4 °C. Droplets were read on a QX600 Droplet Reader (Bio-Rad). The following day, Parallel RPP30 assays were used to correct for DNA shearing and to calculate cell equivalents, as previously described^21^. Samples containing fewer than 7,500 droplets or fewer than 40,000 cell equivalents were excluded. Positive and negative controls were performed in duplicate. QX Manager Software Standard Edition (v.2.1) was used to acquire and analyse data.

### Viral outgrowth assay

For limiting dilution virus culture assays, CD4 T cells were separated from PBMCs using a REAlease CD4 MicroBead Kit (Miltenyi), activated with anti-CD3/CD28-coated microbeads (Gibco) at a bead-to-cell ratio of 1:1 and 30 U ml^–1^ of IL-2 (Peprotech) for 24 h and co-cultured with SupT1-R5 cells or CD4 lymphoblasts in the presence of 100 U ml^–1^ IL-2 in RPMI 1640 GlutaMAX cell culture medium (Gibco) containing 10% heat-inactivated fetal calf serum (Sigma), 100 U ml^–1^ penicillin and 100 µg ml^−1^ streptomycin (both from Biochrom). The lymphoblasts used were prepared from PBMCs of a CCR5 wild-type donor by activation with 1 µg ml^–1^ phytohaemagglutinin (Sigma) and 100 U ml^–1^ of IL-2 for 2 days and subsequent depletion of CD8 and CD56 cells (Miltenyi). Culture supernatants were collected every 2 or 3 days and fresh medium was added. Supernatants were stored at −80 °C until analysis for virus production by quantitative measurement of production of the HIV-1 core protein p24 with a HIV-1 p24 ELISA assay (Sino Biological) according to the manufacturer’s protocol. The infectious HIV units were quantified using the IUPMStats (v. 1.0) calculator with an estimated LLOD of <0.05 (first time point, 62 months after treatment interruption) or 0.006 (second time point, 78 months after treatment interruption) infectious units per million CD4 T cells, respectively^65^.

### CCR5 genotyping and haplotyping

gDNA was extracted from heparinized peripheral blood, fingernail samples or hair follicles using a QIAamp DNA Mini Kit (Qiagen). DNA was then subjected to PCR amplification with oligonucleotides for the *CCR5* gene spanning the Δ32 region from nucleotides 805 to 988 on chromosome 3p21.31 (accession no: NM_000579.4). The expected fragments were 184 bp for the CCR5 wild-type and 152 bp for the CCR5Δ32 variant. Mismatched heteroduplex DNA was formed in samples containing both variants. Haplotyping was performed according to previously described methods^23,66^. The 25 µl PCR reaction mixture consisted of 1 µl DNA (corresponding to 25–100 ng per reaction), 5 µl 5× OneTaq standard reaction buffer, 0.5 µl dNTPs (10 mM), 0.5 µl each of forward and reverse primers (10 µM) (*CCR5* forward GACGAGAAAGCTGAGGGTAAGA, *CCR5* reverse TAACCGTCTGAAACTCATTCCA; *CCR2* forward TACGGTGCTCCCTGTCATAAA; *CCR2* reverse TGGAAAATAAGGGCCACAGAC) and 0.125 µl OneTaq DNA polymerase (NEB, M0480S). The thermal cycling conditions were as follows: initial denaturation at 94 °C for 30 s; 30 cycles of 94 °C for 15 s, 55 °C for 15 s and 68 °C for 90 s (for *CCR5* amplification) or 30 s (for *CCR2* amplification); followed by a final extension at 68 °C for 5 min. The expected PCR product sizes were 1,388 bp for *CCR5* and 504 bp for *CCR2*. Reverse primers were used for Sanger sequencing (Eurofins Genomics). Polymorphisms in the promoter region of *CCR5* (A29G, G208T, G303A, T627C, C630T, A676G and C927T) and in the coding region of *CCR2* (V64I) were determined using SnapGene.

### HIV susceptibility assay

PBMCs from HIV-negative individuals were activated for 3 days with 3 µg ml^–1^ phytohaemagglutinin (Merck) in the presence of 100 U ml^–1^ IL-2 (Merck) for the last 2 days in RPMI 1640 cell culture. Cells were washed and cultured at 10^6^ per ml with HIV-1 strain HTLVIIIB, Ba-L or SF162 or with CXCR4-tropic (isolate 1.2), CCR5-tropic (isolate 2) or dual X4/R5-tropic (isolate 1.1) primary patient isolate from the German HIV-1 Seroconverter Cohort^67^ at a multiplicity of infection of 0.01 in medium supplemented with 100 U ml^–1^ IL-2. Co-receptor usage was confirmed genotypically from HIV-1 population sequences with an ambiguity threshold of 20% using Geno2Pheno[coreceptor]^68^. HIV-1 population sequences were determined as previously described^69^ but using a Nextera XT DNA Library Preparation Kit and a MiSeq Reagent Kit v.3 (Illumina). Viral stocks diluted in cell-free medium served as a background control, the cells alone as a mock control and cell-free virus suspensions as a control for background corrections. Supernatants were removed from cell cultures and cell-free controls as indicated, replaced by fresh medium and stored at −80 °C until analysis for viral replication by quantitative measurement of production of the HIV-1 core protein p24 with an in-house developed HIV-1 p24 antigen capture ELISA that was performed as previously described^70^. In brief, 96-well microtiter plates (Maxisorp, Thermo Scientific) were coated with a previously determined optimal dilution of purified AG3.0 antibody in bicarbonate buffer (pH 9.6) and incubated overnight at 4 °C. Plates were then washed three times with PBS containing 0.05% Tween-20 (PBST) and incubated with blocking buffer (2% milk powder in PBS (PM)) for 45 min at 37 °C in a humidified, 5% CO_2_ incubator. Samples were diluted 1:100, 1:200 or 1:500 in blocking buffer plus 0.05% Tween-20 (PMT). As standard for quantification, a HIV-1 virus lysate previously determined with a commercial ELISA (HIV-1 p24 antigen ELISA, Aalto Bio Reagents) to contain 43.42 p24 ng ml^–1^ was also titrated down the plate. After incubation for 45 min at 37 °C, the plates were washed three times with PBST and incubated with an in-house pooled human anti-HIV-1 sera at a 1:10,000 dilution in PMT. After incubation for 45 min at 37 °C the plates were washed three times with PBST, and goat anti-human IgG–peroxidase conjugate (Sigma-Aldrich) diluted 1:1,000 in PMT was added to all wells. Finally, the plates were washed three times and incubated with 100 μl per well of 3,3′,5,5′-tetramethylbenzidin (Sigma-Aldrich) substrate. After 5–10 min, the desired colour development was achieved and the reaction was stopped by the addition of 1 M H_2_SO_4_ before measuring the optical density at 450 versus 620 nm on an ELISA microplate reader (Infinite 200, Tecan). At day 12, cells were collected and DNA was isolated using a QIAamp DNA Blood Mini Kit (Qiagen). Quantitative provirus PCR was conducted on a CFX96 instrument (Bio-Rad) as previously described^71^.

### Phenotyping characterization of T cells

Samples for CCR5 analyses were processed within 5 min of collection. Heparinized blood was incubated with antibodies for 15 min at room temperature, and after lysis of erythrocytes, cells were immediately analysed by flow cytometry. The following antibodies were used: anti-CD3-APC-H7 (SK7; BD Biosciences, 1:100); anti-CD4-PerCPCy5.5 (SK3; BD, 1:25); anti-CD8-Pacific Blue (RPA-T8; BD, 1:200); anti-CD14-PE-Cy7 (61D3, eBioscience, 1:200); anti-CD19-PE-Cy7 (1:100); CD45RO-VioGreen (UCHL-1; Miltenyi, 1:100); and anti-CCR5-APC (2D7; BD, 1:10). Clone 2D7 recognizes the wild-type but not the Δ32 variant of CCR5, as the 32-bp deletion results in the loss of the 2D7 binding site. CCR5 expression density on CD4 T cells was evaluated as the CCR5 MFI of CCR5 CD4 T cells divided by the MFI value of total CD4 T cells obtained with the corresponding fluorescence-minus-one control and is expressed as the MFI ratio. Analysis of CD4 T cell composition in whole blood was performed as previously described^72^ using the following antibodies: anti-CD4-PerCPCy5.5 (1:35); anti-CD31-PE (WM59; BD, 1:35); anti-CD45RO-APC (UCHL-1; BD 1:450); and anti-CD62L-FITC (Dreg-56; BD, 1:70). Absolute numbers of CD4^+^ and CD8^+^ T cells were determined using TruCount tubes and CD3/CD4/CD8 TritTest (BD) according to the manufacturer’s protocol. Data were acquired on a FACSCanto II system (BD) and analysed with FlowJo software (v.10.10.0; BD).

### V3 loop sequencing and prediction of HIV-1 co-receptor usage

PCR products including the V3 loop region were generated using the primers HIV6945A and HIV7626B. Next-generation sequencing was performed based on semiconductor sequencing on an IonTorrent PGM platform using library prep, emulsion PCR and sequencing reagents from Vela Diagnostics. Resulting sequences were pre-processed using the geno2pheno[454] preprocessor and uploaded to the geno2pheno[454] internet platform^25^. Results were interpreted using cut-off values of 2% population and false positive rate of 3.5%^73^.

### Phylogenetic tree construction

The sequences and phylogenetic tree were constructed using previously described methods^2^. In brief, sequences were aligned using MUSCLE (v.3.8.155)^74^. Two HIV-1 subtype D isolates from the Los Alamos HIV Sequence Database (http://www.hiv.lanl.gov/) were included as the outgroup. Reference sequences from laboratory isolates (HTLVIIIB, Ba-L and SF162) and German HIV-1 Seroconverter Study isolates (isolates 1.1, 1.2 and 2) were also included^67^. Phylogenetic analysis was performed using raxmlGUI (v.2.0)^75^. The maximum likelihood phylogeny was visualized using FigTree (v.1.4.4).

### Human plasma RNA-derived single-genome sequencing

Plasma RNA was isolated using a MinElute Virus Spin Kit (Qiagen), incorporating a DNase I digestion step (Qiagen). cDNA was synthesized with the antisense primer envB3out (5′-TTGCTACTTGTGATTGCTCCATGT) using SuperScript III reverse transcriptase (Thermo Fisher) followed by treatment with RNase H (Thermo Fisher). The resulting *env* cDNA was then amplified using a protocol based on previously established methods^76,77^. PCR amplification was carried out with Phusion Hot Start Flex DNA polymerase (New England Biolabs). The first round of PCR was performed at 98 °C for 45 s, followed by 35 cycles of 98 °C for 15 s, 55 °C for 30 s and 72 °C for 4 min, with a final extension at 72 °C for 15 min. A 1 µl aliquot of the first-round PCR product served as a template for the second-round PCR, which followed similar cycling conditions: 98 °C for 45 s, 45 cycles of 98 °C for 15 s, 55 °C for 30 s and 72 °C for 4 min, ending with 15 min at 72 °C. Purified PCR products were subjected to Sanger sequencing, and sequence data were analysed using Geneious software (Dotmatics).

### Pseudovirus production

Pseudoviruses were prepared in HEK293T cells to produce the autologous patient pseudoviruses B2, Ba-L_26 and YU-2 as previously reported^78^. To generate pseudovirus B2-B7 from single-genome-sequencing-derived *env*, an expression plasmid with the respective *env* sequence was ordered from TWIST Bioscience.

### Serum and plasma IgG isolation for neutralization and binding assays

Serum and plasma samples were initially heat-inactivated at 56 °C for 40 min and either directly used for assessment of neutralizing activity or incubated overnight at 4 °C with Protein G Sepharosebeads (GE Life Sciences) to isolate IgG. The bound antibodies were eluted from the beads using 0.1 M glycine (pH 3.0) and then neutralized with 1 M Tris (pH 8.0). Subsequently, the buffer was exchanged to PBS, and the antibodies were concentrated using Amicon 30 kDa centrifugal filter units (Millipore). The purified IgG samples were stored at 4 °C until they were used in experiments.

### Anti-gp120 and anti-gp140 ELISA

High-binding 96-well ELISA plates (Corning) were coated overnight with purified Env proteins His-tagged clade B YU2 trimeric gp140 and monomeric gp120 in PBS (gift from K. de la Rosa). After washing with PBST, plates were blocked for 1 h with 2% BSA (blocking solution), washed and incubated with 1:5 serially diluted plasma with 2% BSA (1:10 starting dilution). After washing, goat anti-human IgG–peroxidase conjugate (1:10,000, Invitrogen) and 3,3′,5,5′-tetramethylbenzidin (Sigma-Aldrich) substrate were added to the plates. Experiments were performed using a microplate absorbance reader, with absorbance measured at 450 nm. All plasma were tested in duplicate in at least two independent experiments.

### Neutralization assays

Neutralization assays were carried out as previously described^78^. In brief, serial dilutions of plasma or purified IgG samples (3BNC117, VRC01, 1–18, N49P7, VRC07-523-LS, 8ANC195, 10–1074, PGDM 1400, PG16, SF12, 10E8 and MG053, starting concentration of 20 µg ml^–1^) were incubated with pseudoviruses at 37 °C for 1 h. TZM-bl cells were then added at a density of 10^4^ cells per well in a 96-well plate containing 250 µl medium supplemented with 10 µg ml^–1^ DEAE dextran. After incubating for 2 days at 37 °C in a 5% CO_2_ environment, 150 µl of culture supernatant was removed, and 100 µl of luciferase substrate was added. Luminescence was measured with a luminometer following a brief 2 min incubation. Background luminescence from non-infected TZM-bl cells was subtracted to determine relative luminescence units (RLUs). The inhibitory concentration (IC_50_) and half-maximal inhibitory dilution (ID_50_) values were calculated based on the antibody or IgG concentrations and plasma dilutions, respectively, required to achieve 50% inhibition compared with virus-only controls. To test for non-specific activity of the plasma or isolated IgG samples, pseudotyped viruses based on murine leukaemia virus were included. All samples were tested in duplicate. Bioluminescence was assessed using a luciferin/lysis buffer (composed of MgCl_2_, ATP, coenzyme A, IGEPAL and d-luciferin). Luminescence was measured with a luminometer (Berthold TriStar^2^S). Data were analysed using Microsoft Excel for Mac (v.14.7.3). Statistical analysis was performed using GraphPad Prism (v.9.3.1).

### Virus-specific whole-blood stimulation

Heparinized venous blood was stimulated with mixtures of peptides consisting of 15-mer sequences with 11 amino acid overlap originating from the Gag, Pol, Nef or Env protein of HIV (all jpt Technologies), the BZLF1, EBNA-1 or LMP1 protein of EBV (all Miltenyi Biotec), the pp65 (jpt) or IE-1 (Miltenyi) of CMV, or the U90 protein of HHV-6 (jpt). For this, 1 ml of blood was incubated in the presence of 1 µg ml^–1^ of each co-stimulatory antibody to CD28 and CD49d (BD Biosciences) with 0.6 nmol of each peptide at 37 °C, 5% CO_2_ for 6 h. Brefeldin A (Sigma) at 10 µg ml^–1^ and monensin (GolgiStop; BD) at 2 µg ml^–1^ were added 2 h after the start of incubations. As a positive control, cells were incubated with 2 µg ml^–1^ SEB (Sigma), and 3–6 samples without peptides served as background controls in each assay. Following stimulation, blood was treated with 2 mM EDTA for 10 min and erythrocytes were lysed with RBC lysis buffer (BioLegend). Cells were washed, and virus-specific T cells were then identified by intracellular cytokine staining. For this, cells were labelled with surface antibodies, then fixed and permeabilized using a Cytofix/Cytoperm Kit (BD), labelled with antibodies against intracellular cytokines and immediately analysed by flow cytometry. The following antibodies were used: anti-CD3-APC-H7 (1:50), anti-CD4-FITC (1:30), anti-CD8-Pacific Blue (1:100), anti-CD14-PE-Cy7 (1:100) and anti-CD19-PE-Cy7 (1:50) for surface antigens; and anti-IL-2-Brilliant Violet 510 (5344.111; BD, 1:20), anti-IFNγ-APC (B27; BD, 1:100) and anti-TNF-PE (Mab11; BD, 1:200) for intracellular staining. Purified human immunoglobulin at 3 mg ml^–1^ (CSL Behring) was added for elimination of non-specific binding. Lymphocytes were gated based on doublet discrimination and characteristic forward and side scatter properties. CD8 or CD4 T cells were identified by selecting cells that were negative for CD14 and CD19 (excludes monocytes and B cells) and positive for CD3 and either CD8 or CD4. Between 100,000 and 500,000 CD4 or CD8 T cell events were collected per sample.

### Virus-specific CD8 T cell degranulation assay

PBMC stimulations were performed as described for whole blood except that PBMCs at a density of 10^6^ per ml were incubated for 5.5 h, and brefeldin A and monensin were added together with anti-CD107a-PerCP-Cy5.5 (H4A3; BioLegend, 1:200) at the start of incubation. The following additional antibodies were used for flow cytometry analysis: anti-CD3-APC-H7 (1:50), anti-CD8-Pacific Blue (1:100), anti-CD14-PE-Cy7 (1:100), anti-CD16-FITC (3G8; BD, 1:50), anti-CD19-PE-Cy7 (1:50), anti-CD56-FITC (MEM-188; BioLegend, 1:50), anti-IFNγ-APC (1:100) and anti-TNF-PE (1:200). CD8 T cells were identified by selecting cells negative for CD14, CD16, CD19 and CD56 (excludes monocytes, B cells and NK cells) and positive for both CD3 and CD8. Between 20,000 and 50,000 CD8 T cell events were collected per sample.

### KIR genotyping

Typing for KIR was performed using an in-house-developed high-throughput approach at DKMS Life Science Laboratory^79^. Using short amplicons obtained from Illumina sequencers, targeted sequences comprised exons 3, 4, 5, 7, 8 and 9. Based on calibrated read counts, copy number and allelic variants were determined per exon in reference to the IPD-KIR database. Final results were derived by disentangling gene-bridging cases, when different KIR genes share identical exonic sequences. To avoid loss of information in ambiguous cases, results are reported using GL-strings^80^.

### NK cell enrichment and cell culture

NK cells were enriched from PBMCs using a negative-selection strategy with an EasySep Human NK Cell Enrichment Kit (Stemcell Technologies) according to the manufacturer’s protocol. Isolated NK cells were cultured in RPMI supplemented with 10% (v/v) fetal bovine serum (R10), 1% penicillin–streptomycin (Sigma Aldrich) and 1% GlutaMAX (Gibco). Where indicated, 1 ng ml^−1^ IL-15 (Peprotech) alone or in combination with 50 U ml^–1^ IL-2 (Novartis) was added. NK cells were then cultured overnight at 37 °C in 5% CO_2_. Raji cells were cultured in R10 with 1% GlutaMAX and 1% penicillin–streptomycin.

### Phenotyping of NK cells

Cryopreserved PBMCs from HIV-1-negative donors (*N* = 7) and from donor B2 were split into two panels after staining with a Zombie Aqua Fixable Viability Kit (BioLegend, 1:50) for 10 min in the dark at 4 °C followed by 10 min of incubation with human Fc-block (BD Bioscience, 1:50) at room temperature. To analyse KIR expression (panel A), PBMCs were stained with anti-KIR3DL1-FITC (DX9; BioLegend, 1:100), anti-KIR2DL1-APC Vio770 (REA284; Miltenyi Biotec, 1:25), anti-CD3-AF700 (UCHT1; BD Bioscience, 1:100), anti-NKG2C-APC (REA205; Miltenyi Biotec, 1:100), anti-CD56-BV786 (NCAM16.2; BD Bioscience, 1:100), anti-KIR2DL2/DL3-biotin (DX27, Miltenyi Biotec, 1:10), anti-CD14-BV510 (M5E2; BioLegend, 1:50), anti-CD19-BV510 (HIB19; BioLegend, 1:50), anti-CD57-pacific blue (HNK-1; BioLegend, 1:100), anti-NKG2A-PE-Cy7 (Z199; Beckman Coulter, 1:100) and anti-KIR3DL1/DS1-PE (REA168; Miltenyi Biotec, 1:50). To analyse adaptive NK cell subsets (panel B), PBMCs were stained with anti-CD7-PerCP-Cy5.5 (4H9/CD7; BioLegend, 1:100), anti-Siglec7-APC-Vio770 (REA214; Miltenyi Biotec, 1:25), anti-CD2-AF700 (RPA-2.10; BioLegend, 1:100), anti-NKG2C-APC (REA205; Miltenyi Biotec, 1:100), anti-CD16-BV785 (3G8; BioLegend, 1:100), anti-ILT2-biotin (REA998; Miltenyi Biotec, 1:50), anti-CD161-BV605 (HP-3G10; BioLegend, 1:100), anti-CD8-BV570 (RPA-T8; BioLegend, 1:100), anti-CD14-BV510 (M5E2; BioLegend, 1:50), anti-CD19-BV510 (HIB19; BioLegend, 1:50), anti-CD57-pacific blue (HNK-1; BioLegend, 1:100), anti-NKG2A-PE-Vio770 (REA110; Miltenyi Biotec, 1:100), anti-CD56-BUV737 (NCAM16.2; BD Bioscience, 1:100) and anti-CD3-BUV395 (UCHT1; BD Bioscience, 1:100).

Cells stained with either panel were incubated for 30 min in the dark at 4 °C, washed twice with 1% FBS–PBS and then incubated with Streptavidin BV711 (BioLegend, 1:200) for 20 min in the dark at 4 °C. After washing twice with 1% FBS–PBS, cells stained with panel A were left unfixed and immediately analysed using a BD FACSAria. For panel B, intracellular staining was performed using a BD Cytofix/Cytoperm Fixation/Permeabilization Kit (BD Biosciences), along with anti-FcεR1γ-FITC (polyclonal, Merck, 1:50) and anti-PLZF-PE (Mags.21F7; eBioscience, 1:100) according to the manufacturer’s protocol. Cells were washed and stored in 1% FBS–PBS at 4 °C until analysis on a Cytek Aurora flow cytometer.

For phenotyping of NK cell subsets of patient B2 that degranulated in response to rituximab, cells were first incubated with a Zombie Aqua Fixable Viability Kit (BioLegend, 1:50) for 10 min at 4 °C in the dark. After washing, surface staining was performed for 20 min at 4 °C in the dark using the following antibodies: anti-NKG2C-APC (REA205; Miltenyi Biotec, 1:100), anti-NKG2A-PE Vio770 (REA110; Miltenyi Biotec, 1:100), anti-CD16-BV785 (3G8; BioLegend, 1:100), anti-Siglec7-APC-Vio770 (REA214; Miltenyi Biotec, 1:25), anti-CD7-PerCP-Cy5.5 (4H9/CD7; BioLegend, 1:100), anti-CD56-BUV395 (NCAM16.2; BD Biosciences, 1:100), anti-KIR3DL1-AF700 (DX9; BioLegend, 1:100), anti-KIR2DL2/DL3-BV711 (DX27; BD Biosciences, 1:50), anti-KIR2DL1-PE (HP-DM1; BioLegend, 1:50) and anti-CD57-PE Dazzle 594 (HNK-1; BioLegend, 1:100). Next, cells were washed twice with 1% FBS–PBS, fixed with 1× CellFIX (BD Biosciences) and analysed on a Cytek Aurora flow cytometer. For the ADCC assay using immobilized Env to assess the activity of plasma from patient B2, NK cells were incubated at room temperature for 20 min with a Live/Dead Fixable Near-IR Dead Cell Stain Kit (Invitrogen, 1:1,000) together with anti-CD16 BV711 (3G8; BioLegend, 1:100) and anti-CD56 BUV395 (NCAM16.2; BD Biosciences, 1:100). After staining, cells were washed twice with PBS containing 1% FBS, fixed with 4% paraformaldehyde (Sigma-Aldrich) and analysed using a LSR Fortessa flow cytometer (BD Biosciences). Flow cytometry data were analysed using FlowJo (v.10.8.1; BD Life Sciences) with the UMAP and FlowSOM plugin.

### Dimensionality reduction analysis of NK cells

The flow cytometry data (panel B) were gated on NK cells before being subjected to dimensionality reduction analysis in R (v.4.4.1). The analysis was based on the publicly available R package Seumetry (v.0.1.0)^81^. Fcs files from patient B2 and seven HIV-negative individuals (as the control group) were combined into a flowset and then converted into a Seurat object before being downsampled to approximately 20,000 cells per donor and transformed using the arcsinh transformation method. After manually removing outliers, scaling the data and performing a principal component analysis, a UMAP based on the first ten principal components was generated. The final NK cell clusters were identified using the Louvain algorithm, followed by merging clusters with similar identities.

### ADCC assays

To evaluate the ADCC capacity of different NK cell subsets from patient B2, enriched NK cells were cultured overnight in R10 supplemented with 1% penicillin–streptomycin, 1% GlutaMAX and 1 ng ml^–1^ IL-15. On the next day, cells were transferred into a U-bottom 96-well plate. Before co-incubation, Raji cells were labelled with the fluorescent dye CFSE (Invitrogen) by incubating them with CFSE (1:2,000) for 8 min in 1 ml PBS at room temperature, with mixing after 4 min. The reaction was quenched using 7 ml 10% FBS–RPMI. Subsequently, Raji cells (10^6^ cells per ml) were coated with 30 µg ml^–1^ rituximab (Invivogen) for 30 min at 37 °C in 5% CO_2_. Uncoated Raji cells were used as a control. NK and Raji cells were co-cultured at an E:T ratio of 2:1 for 3.5 h at 37 °C in 5% CO_2_ in the presence of anti-CD107a-BV421 (H4A3, BioLegend, 1:100). After co-culture, cells were stained for flow cytometry analysis (see the section ‘Phenotyping of NK cells’). To assess ADCC activity induced by plasma from patient B2, Env protein was immobilized onto non-tissue-culture-treated, flat-bottom 96-well plates (Corning). Wells were either coated overnight at 4 °C with 10 µg ml^–1^ HIV-1 Ba-L_01 gp120 protein (Sino Biological) or left uncoated (PBS only, as control). The following day, wells were washed three times with PBS and blocked with 2% BSA (Sigma-Aldrich) in PBS overnight at 4 °C. After blocking, wells were washed with PBS and incubated for 45 min at 4 °C with 60 µl per well of one of the following: 10 µg ml^−1^ of the three broadly neutralizing antibodies in PBS, including 1–18, VRC01 and 10–1074; 1:1,000 PBS-diluted plasma samples from five elite controllers and five longitudinal time points from patient B2; 1:1,000 PBS-diluted plasma from an individual who was HIV-negative; or PBS only (negative control). Wells were then washed twice with PBS. PBMCs from heterologous HIV-1-negative donors were isolated 1 day before the assay, and NK cells were enriched and cultured in R10 supplemented with 1% penicillin–streptomycin, 1% GlutaMAX, 1 ng ml^–1^ IL-15 and 50 U ml^–1^ IL-2 overnight. Next, 10^5^ NK cells were added to the coated wells in a volume of 100 µl, centrifuged at 300*g* for 3 min (acceleration and deceleration settings of 3) and incubated for 4 h at 37 °C with 5% CO_2_ in the presence of anti-CD107a BV421 antibody (H4A3; BioLegend, 1:100). Following incubation, wells were thoroughly washed with MACS buffer (Miltenyi Biotec) to detach NK cells, and cells were transferred to 96-well U-bottom plates for flow cytometry analysis (see the section ‘Phenotyping of NK cells’).

### Models for rebound time and probability

A previous study^35^ defined a mechanistically motivated, phenomenological model that describes both short-term and long-term viral rebounds using data from a large cohort of* N* = 235 ATI participants from the AIDS Clinical Trials Group (ACTG) studies (371, A5024, A5068, A5170, A5187 and A5197)^35^.

Inclusion criteria were suppressive ART such that HIV-1 RNA was <50 copies per ml and that participants had received no other immunological interventions. To properly model the breadth of individuals, inclusive of some who rebounded months after ATI, the authors applied careful model selection theory and arrived at a model whereby reactivation rate decreases with time from an early rate ${r}_{0}$ to a long-term rate ${r}_{\infty }$, that is still a piecewise function:

1 $$r(s)=0\,\mathrm{when}\,s\le \tau $$

2 $$r(s)={r}_{\infty }+({r}_{0}-{r}_{\infty })\exp [-k\times (s-\tau )],{\rm{else}}$$

With a typical hazard function assumption that rebound probability depends on the exponential of the cumulative probability of this function over the time, the ultimate probability of rebound is written (as shown in Fig. 5a)

3 $$P(t)=1-\exp \left[-{\int }_{\tau }^{t}r(s){\rm{d}}s\right]$$

Note, in this and other data-validated approaches, the model requires a delay of approximately 5 days, such that rebound probability function ${p}_{\mathrm{VR}}$ is defined as a piecewise function where its value is zero before the delay time $\tau$. We opted to notate this function using a Heaviside step function $\Theta (y)$ whose value is 1 when $y > 0$ and 0 otherwise. Therefore, we wrote the general time-dependent rebound probability.

4 $${p}_{\mathrm{VR}}(t)=\Theta (\tau -t)P(t)$$

Where $P(t)$ is the function governing rebound after the delay time, here equation (3).

Estimated parameters were with mean and (95% CI) values: *r*_0_ = 0.088 (0.073, 0.106) per day, *r*_∞_ = 0.002 (0.001, 0.004) per day, *k* = 0.029 (0.022, 0.040) per day and *τ* = 4.98 (4.58, 5.41) days.

We calculated the probability of not rebounding for our patient ATI using these parameters in equation (4), evaluated at *t* = 6 years.

Next, we also took a more classic model of viral rebound with a constant recrudescence rate and explored what values this constant rate ${r}_{\Delta \pm }$ would be required to explain no rebound over the entire period for this HCT patient with heterozygous CCR5Δ32. We estimated the value of ${r}_{\Delta \pm }$ by simply solving for the values that produce a $P$ = 5, 50 and 95% chance of rebound in 6 years, that is, $\mathrm{ln}(1-P)/-6\,\mathrm{year}={r}_{\Delta \pm }.$

### Data presentation

Data figures were arranged in Affinity Designer and Adobe Illustrator. The maximum likelihood phylogeny was visualized using FigTree (v.1.4.4).

### Reporting summary

Further information on research design is available in the Nature Portfolio Reporting Summary linked to this article.

## Online content

Any methods, additional references, Nature Portfolio reporting summaries, source data, extended data, supplementary information, acknowledgements, peer review information; details of author contributions and competing interests; and statements of data and code availability are available at 10.1038/s41586-025-09893-0.

## Supplementary information

Supplementary Fig. 1 NK cell gating strategy.

Reporting Summary

Supplementary Table 1 Clinical immunophenotyping results of patient B2 during the pre-transplant period.

Supplementary Table 2 List of potential GVL-mediating peptide variants present in patient B2.

Supplementary Table 3 List of CD4 T cell-related miHA peptide variants present in patient B2.

Supplementary Table 4 List of Y chromosomal miHA peptide variants present in patient B2.

## Figures and Tables

**Extended Data Table 1:**
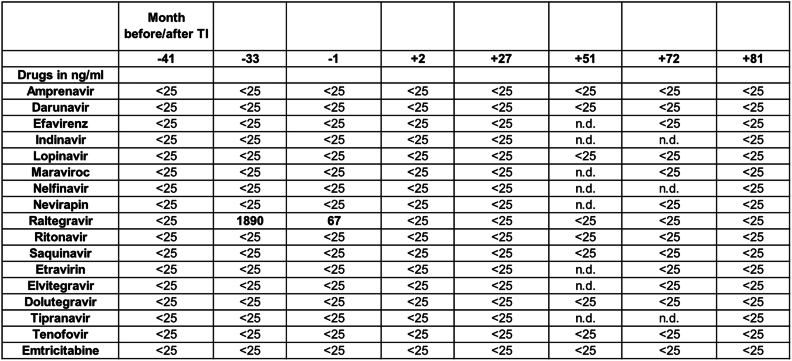
Antiretroviral Drug Screening

**Extended Data Table 2:**
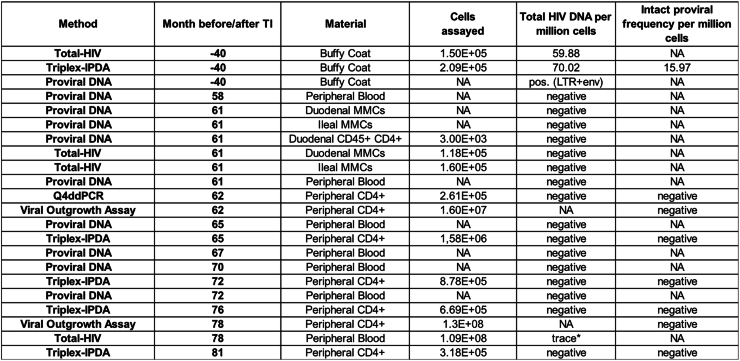
Reservoir measurements

HIV reservoir measurements were conducted in peripheral blood, blood-derived CD4^+^ cells, and gut-associated lymphoid tissue at various time points before and after antiretroviral treatment interruption, ATI. The intact viral reservoir was assessed by either viral outgrowth assay, Triplex IPDA, or Q4ddPCR. For Triplex IPDA, genomic DNA from isolated CD4^+^ cells was analyzed using digital PCR. Triplex IPDA targets regions in the env gene and the packaging signal to determine proviral intactness, alongside RU5 to quantify total HIV DNA. Proviruses that were positive for both *env* and packaging signal were detected prior to stem cell transplantation but not after transplantation. Q4ddPCR involved simultaneous targeting of four genomic regions (env, gag, pol and packaging signal) in a droplet digital PCR platform. No positive droplets were detected for any of these targets. For the viral outgrowth assay, a total of 1.7×10^7^ CD4^+^ cells were stimulated. Supernatants were tested for viral production using p24 ELISA, without detection of infectious units. Additionally, total HIV DNA in gastrointestinal tissue was measured via digital PCR, targeting the RU5 region. Genomic DNA was extracted from mononuclear cells in the lamina propria of the jejunum or duodenum. Duplicate measurements were performed on four independent biopsies, with no positive partitions detected. Proviral DNA was quantified from peripheral blood or intestine-derived DNA using two independent quantitative PCR reactions, targeting the *env* gene or the 5′ long terminal repeat region. NA, not applicable, MMCs, mucosal mononuclear cells.

## Data Availability

The data supporting the findings of this study are provided in the main figures and supplementary materials of the Article. Source data will be made available upon request to the corresponding authors. Viral sequences have been deposited into GenBank (https://www.ncbi.nlm.nih.gov/genbank/) with the accession codes PQ768542–PQ768825.

## Code availability

Computer code to perform the mathematical model of viral rebound is available at GitHub (https://github.com/dbrvs/B2rebound/tree/main). Dimensionality reduction analysis of NK cells based on the publicly available R package Seumetry (v.0.1.0) is available at GitHub (https://github.com/imsb-uke/Seumetry).
