# Supplementary material for: Sustained HIV-1 remission after heterozygous CCR5Δ32 stem cell transplantation
Source: Nature. 2025 Dec 1;650(8102):701–9. doi: 10.1038/s41586-025-09893-0 (PMC12916306; doi:10.1038/s41586-025-09893-0)
Supplement: Supplementary file 1 — NK cell gating strategy. [file 41586_2025_9893_MOESM1_ESM.pdf]

nature portfolio

https://doi.org/10.1038/s41586-025-09893-0

Supplementary information

Sustained HIV-1 remission after heterozygous CCR5Δ32 stem cell transplantation

In the format provided by the authors and unedited

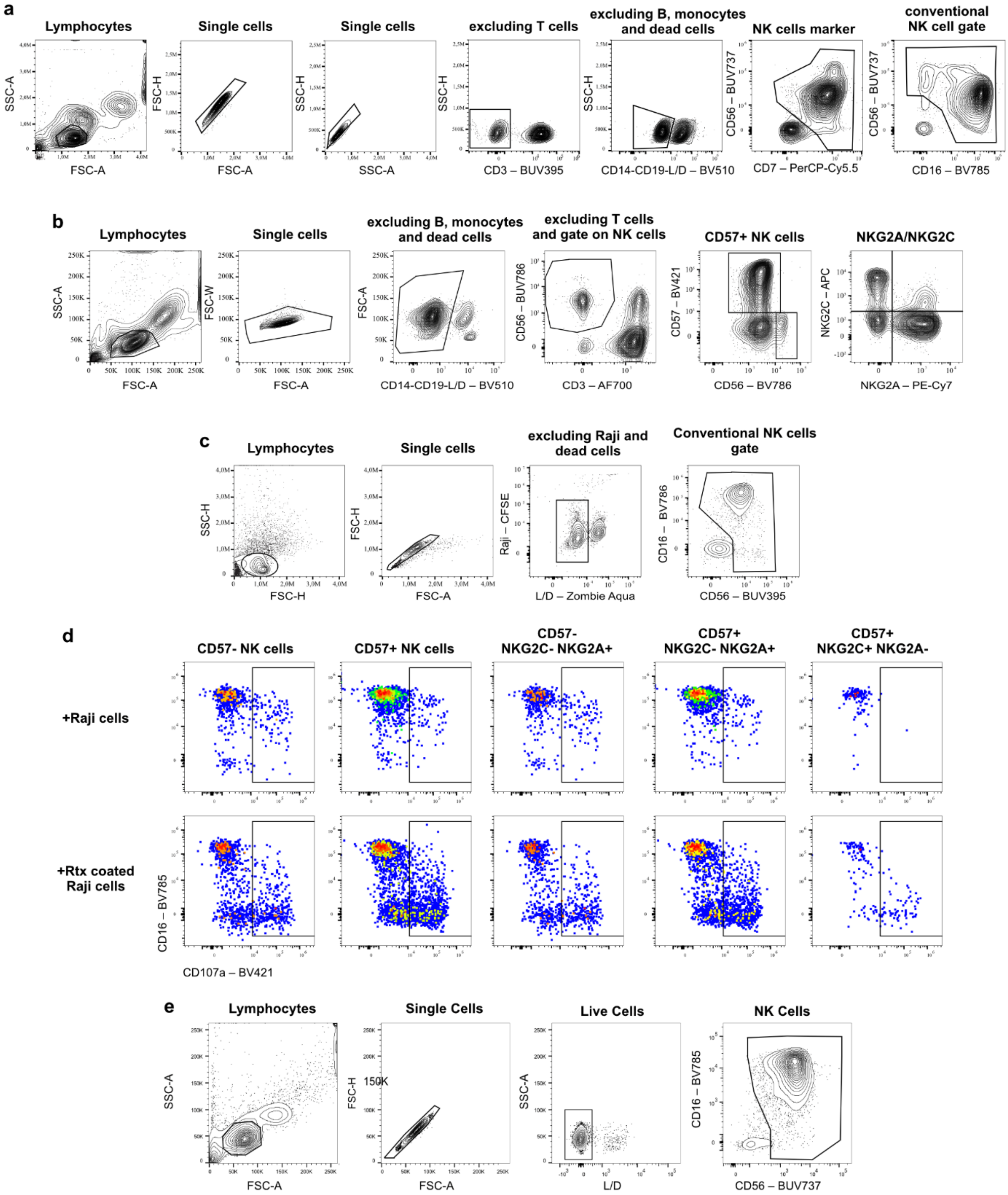
a
Lymphocytes
Single cells
Single cells
excluding T cells
excluding B, monocytes and dead cells
NK cells marker
conventional NK cell gate
SSC-A
FSC-A
FSC-H
FSC-A
SSC-H
SSC-A
SSC-H
CD3 – BUV395
SSC-H
CD14-CD19-L/D – BV510
CD56 – BUV737
CD7 – PerCP-Cy5.5
CD56 – BUV737
CD16 – BV785
b
Lymphocytes
Single cells
excluding B, monocytes and dead cells
excluding T cells and gate on NK cells
CD57+ NK cells
NKG2A/NKG2C
SSC-A
FSC-A
FSC-W
FSC-A
FSC-A
CD14-CD19-L/D – BV510
CD56 – BUV786
CD3 – AF700
CD57 – BV421
CD56 – BV786
NKG2C – APC
NKG2A – PE-Cy7
c
Lymphocytes
Single cells
excluding Raji and dead cells
Conventional NK cells gate
SSC-H
FSC-H
FSC-H
FSC-A
Raji – CFSE
L/D – Zombie Aqua
CD16 – BV786
CD56 – BUV395
d
CD57- NK cells
CD57+ NK cells
CD57- NKG2C- NKG2A+
CD57+ NKG2C- NKG2A+
CD57+ NKG2C+ NKG2A-
+Raji cells
+Rtx coated Raji cells
CD16 – BV785
CD107a – BV421
e
Lymphocytes
Single Cells
Live Cells
NK Cells
SSC-A
FSC-A
FSC-H
FSC-A
SSC-A
L/D
CD16 – BV785
CD56 – BUV737

Supplementary Figure 1: NK cells gating strategy.

a, Lymphocytes were gated based on FSC-A/SSC-A. Subsequently, single cells were identified using SSC-H/SSC-A and FSC-H/FSC-A. T cells (CD3+) were then excluded followed by exclusion of B cells (CD19+), monocytes (CD14+) and dead cells. NK cells were first identified by their CD7 and CD56 expression and thereafter conventional NK cell gate was set using CD16 and CD56 expression (Fig.4a,4c). **b,** Lymphocytes were gated based on FSC-A/SSC-A, followed by a single cell gate using FSC-A/FSC-H. B cells, monocytes and dead cells were excluded. NK cells were identified by their lack of CD3 and expression of CD56. CD57 was gated on CD56dim cells, followed by identification of NKG2A and NKG2C expression (Fig. 4b). **c,** Lymphocytes were gated based on FSC-H/SSC-H. Single cells were identified using FSC-A/FSC-H. CFSE+ Raji cells and dead NK cells were excluded. NK cells were identified by their expression of CD16 and CD56. CD57, NKG2A and NKG2C were gated as shown in (b) (Extended Data Figure 7d,e). **d,** Dot plots depict CD107a expression in indicated subset (above) after co-incubation with Raji cells and rituximab-coated Raji cells (Extended Data Figure 7e, f). **e,** Lymphocytes were gated based on FSC-A/SSC-A. Subsequently, single cells were identified using FSC-H/FSC-A. Dead cells were excluded. NK cells were identified by CD16 and CD56 expression.
