# Supplementary material for: Sustained HIV-1 remission after heterozygous CCR5Δ32 stem cell transplantation
Source: Nature. 2025 Dec 1;650(8102):701–9. doi: 10.1038/s41586-025-09893-0 (PMC12916306; doi:10.1038/s41586-025-09893-0)
Supplement: Supplementary file 2 — Reporting Summary [file 41586_2025_9893_MOESM2_ESM.pdf]

nature portfolio

Corresponding author(s): Christian Gaebler

Last updated by author(s): Oct 8, 2025

Reporting Summary

Nature Portfolio wishes to improve the reproducibility of the work that we publish. This form provides structure for consistency and transparency in reporting. For further information on Nature Portfolio policies, see our Editorial Policies and the Editorial Policy Checklist.

Statistics

For all statistical analyses, confirm that the following items are present in the figure legend, table legend, main text, or Methods section.

n/a	Confirmed	
☐	☒	The exact sample size (*n*) for each experimental group/condition, given as a discrete number and unit of measurement
☒	☐	A statement on whether measurements were taken from distinct samples or whether the same sample was measured repeatedly
☐	☒	The statistical test(s) used AND whether they are one- or two-sided<br>*Only common tests should be described solely by name; describe more complex techniques in the Methods section.*
☒	☐	A description of all covariates tested
☒	☐	A description of any assumptions or corrections, such as tests of normality and adjustment for multiple comparisons
☐	☒	A full description of the statistical parameters including central tendency (e.g. means) or other basic estimates (e.g. regression coefficient) AND variation (e.g. standard deviation) or associated estimates of uncertainty (e.g. confidence intervals)
☐	☒	For null hypothesis testing, the test statistic (e.g. *F*, *t*, *r*) with confidence intervals, effect sizes, degrees of freedom and *P* value noted<br>*Give P values as exact values whenever suitable.*
☒	☐	For Bayesian analysis, information on the choice of priors and Markov chain Monte Carlo settings
☒	☐	For hierarchical and complex designs, identification of the appropriate level for tests and full reporting of outcomes
☒	☐	Estimates of effect sizes (e.g. Cohen's *d*, Pearson's *r*), indicating how they were calculated

Our web collection on statistics for biologists contains articles on many of the points above.

Software and code

Policy information about availability of computer code

Data collection

BertholdTech TriStar2S and associated software ICE, Version 1.0.10.0
Qiacuity Software Suite 2.5.0.1
FACSCanto II cytometer (BD Biosciences)
BD LSRFortessa (BD Biosciences)
Cytex Aurora 5 Laser (Cytek Bioscience)
FACSJazz cell sorter cytometer (BD Biosciences)
BD FACS Fusion (BD Biosciences)

Data analysis

geno2pheno[454] at https://454.geno2pheno.org/
GeneMapper Version 3.7 (Thermo Fisher Scientific, Germany)
QX Manager Software Standard Edition, Version 2.1
GraphPad Prism v10
Microsoft Excel for Mac (v14.7.3)
Qiacuity Software Suite 2.5.0.1
FlowJo version 10.10.0 (BD)
FlowJo V10.8.1 including FlowSOM (v4.1.0) and UMAP (v4.1.1) plugin.R (v4.4.1) and R Studio (v2024.04.0) using the Seumetry package (v0.1.0) including all dependencies. The following packages were individually loaded: ggplot2 (v3.5.1), dplyr (v1.1.4), ggcyto (v1.33.0), edgeR (v4.3.16), flowWorkspace (v4.17.0), RBGL (v1.81.0), ncdfFlow (v2.51.0). The packages devtools (v2.4.5), cCustomize (v2.1.2) and svglite (v2.1.3)
Computer code to perform mathematical model of viral rebound is available under https://github.com/dbrvs/B2rebound/tree/main
Dimensionality reduction analysis of NK cells based on the publicly available R package “Seumetry” (v0.1.0) under https://github.com/imsb-

uke/Seumetry
Snapgene (v8.0.2)
CD4 T cell filter for prediction of candidate minor histocompatibility antigens was generated based CD4 T cells in healthy donor PBMC datasets (https://atlas.fredhutch.org/nygc/multimodal-pbmc/)

For manuscripts utilizing custom algorithms or software that are central to the research but not yet described in published literature, software must be made available to editors and reviewers. We strongly encourage code deposition in a community repository (e.g. GitHub). See the Nature Portfolio guidelines for submitting code & software for further information.

Data

Policy information about availability of data

All manuscripts must include a data availability statement. This statement should provide the following information, where applicable:

- Accession codes, unique identifiers, or web links for publicly available datasets
- A description of any restrictions on data availability
- For clinical datasets or third party data, please ensure that the statement adheres to our policy

The data supporting the findings of this study are provided in the main figures and supplementary materials of the article. Source data will be made available upon request to the corresponding authors. Viral sequences have been deposited in GenBank with the accession codes PQ768542 to PQ768825 (https://www.ncbi.nlm.nih.gov/genbank/).

Research involving human participants, their data, or biological material

Policy information about studies with human participants or human data. See also policy information about sex, gender (identity/presentation), and sexual orientation and race, ethnicity and racism.

Reporting on sex and gender	Sex was not considered in this study, as it focused on the characterization of a single case (male).<br>Study participants NK experiments: three female, four male; no information on gender available
Reporting on race, ethnicity, or other socially relevant groupings	These parameters were not considered for this report of a single individual.
Population characteristics	This study presents the case of a male participant, who is 60 years old at the time of reporting. Detailed information on his HLA genotype, as well as his relevant diagnostic and therapeutic history, is provided.<br>NK experiments: Healthy human research participants were chosen based on their CMV status from a large healthy cohort irrespective of genetic background, sex and age. Age ranged from 37-67 (median: 54).
Recruitment	Control individuals were recruited at the Charité Universitätsmedizin Berlin and University Medical Center Hamburg-Eppendorf after providing informed written consent. No self-selective bias was introduced.
Ethics oversight	Written informed consent was obtained from patient B2 following consultation with the local Ethics Committee of Charité Universitätsmedizin Berlin. Biological samples from patient B2 were used for research purposes in accordance with the Ethics Committee of Charité Universitätsmedizin Berlin (reference number EA4/261/23). Control participants, including HIV-negative blood donors, allo-SCT patients and individuals living with HIV, were enrolled at Charité Universitätsmedizin Berlin and University Medical Center Hamburg-Eppendorf under approved ethical protocols (Ethics Committee of Charité Universitätsmedizin Berlin, reference numbers EA2/077/23 and EA4/255/23; and Ärztekammer Hamburg, reference number PV4780). Written informed consent was obtained from all participants, and the studies were conducted in accordance with Good Clinical Practice. HIV isolates from study patients enrolled in the German HIV-1 Seroconverter Study were used. The study was approved by the ethical committee of the Charité University Medicine Berlin (first approval EA2/105/05 with last amendment EA2/024/21).

Note that full information on the approval of the study protocol must also be provided in the manuscript.

Field-specific reporting

Please select the one below that is the best fit for your research. If you are not sure, read the appropriate sections before making your selection.

☒ Life sciences ☐ Behavioural & social sciences ☐ Ecological, evolutionary & environmental sciences

For a reference copy of the document with all sections, see nature.com/documents/nr-reporting-summary-flat.pdf

Life sciences study design

All studies must disclose on these points even when the disclosure is negative.

Sample size	Sample size calculation was not applicable, as this study focused on a single individual.<br>For NK-cell phenotyping, sample size calculation was not applicable as this study was focused on one specific individual, similar to previously published literature (https://doi.org/10.1038/s41591-024-03277-z). Accordingly, sample size calculation for ADCC assays was not applicable

	as the assay was focused on longitudinal sampling from one specific individual. The number of biological replicates was chosen based on previous experience and standards in the field.
Data exclusions	There was no data exclusion.<br>NK experiments: One CMVpos control was excluded due to absent NKG2C staining, suggesting genetic presence of an NKG2C deletion. Therefore, this donor was not suited as a control and excluded from all analysis as pre-established. This donors is already excluded from the population characteristics in the reporting summary above.
Replication	Samples from different time points (biological replicates) were analyzed in all experiments, with the exception of HIV-DNA measurements in gut biopsies. Technical triplicates were performed for viral suppression assays, neutralization assays, and CD4+ T cell susceptibility to HIV-1 infection, while duplicates were used for antibody titration. All replication attempts yielded consistent results.<br>NK experiments: The presence of NKG2A, CD57, and NKG2C subsets in both B2 and HIV-1 negative controls was confirmed using two independent staining panels applied on the same day, with overlapping markers to ensure reproducibility. In addition, ADCC assays were performed with PBMCs from four independent HIV-1 negative donors on the same day; all four biological replicates showed similar results. All attempts at replication were successful.
Randomization	Not applicable, as the study focused on a single case.
Blinding	Not applicable, as the study focused on a single case.

Reporting for specific materials, systems and methods

We require information from authors about some types of materials, experimental systems and methods used in many studies. Here, indicate whether each material, system or method listed is relevant to your study. If you are not sure if a list item applies to your research, read the appropriate section before selecting a response.

Materials & experimental systems

n/a	Involved in the study	
☐	☒	Antibodies
☐	☒	Eukaryotic cell lines
☒	☐	Palaeontology and archaeology
☒	☐	Animals and other organisms
☒	☐	Clinical data
☒	☐	Dual use research of concern
☒	☐	Plants

Methods

n/a	Involved in the study	
☒	☐	ChIP-seq
☐	☒	Flow cytometry
☒	☐	MRI-based neuroimaging

Antibodies

Antibodies used

HRP-conjugated Goat anti-human IgG (Invitrogen, A18805, Lot number 94-118-111423)
goat Anti-Human IgG-Peroxidase antibody (Sigma-Aldrich, A6029, Lot number 0000480816)
Monoclonal anti-HIV-1 Env 3BNC117 (NIH ARP Cat#12474; RRID: AB_2491033)
Monoclonal anti-HIV-1 Env VRC01 (NIH ARP Cat#12033; RRID: AB_2491019)
Monoclonal anti-HIV-1 Env 561_01_18 (Schommers et al, Cell 2020. Genbank Accession no heavy chain: MN867953.1, light chain: MN868009.1)
Monoclonal anti-HIV-1 Env VRC07-523-LS (NAID, NIH. Source Wu et al., 2010. Genbank Accession no heavy chain: HQ654833, light chain: HQ654834)
Monoclonal anti-HIV-1 Env N49P7 (University of Maryland, source Sok et al., 2014)
Monoclonal anti-HIV-1 Env 8ANC195 (Scheid et al, 2011 - RRID: AB_2491037)
Monoclonal anti-HIV-1 Env 10-1074 (NIH ARP Cat#12477; RRID: AB_2491062)
Monoclonal anti-HIV-1 Env PGDM1400 (Sok et al, 2014. Genbank Accession no heavy chain: KJ734532, light chain: KJ734533)
Monoclonal anti-HIV-1 Env PG16 (Walker et al 2009. Genbank Accession no heavy chain: GQ153862, light chain: GQ153861)
Monoclonal anti-HIV-1 Env SF12 (Schoofs et al, 2019. Genbank Accession no heavy chain: MN011123, light chain: MN011124)
Monoclonal anti-HIV-1 Env 10E8 (Huang et al, 2012. Genbank Accession no heavy chain: JQ412634, light chain: JQ412635)
Monoclonal Antibody MG053 (University of Maryland, source Wardemann et al, 2013)
anti-CD28 (L293), anti-CD49d (L25) (Fast Immune, BD Biosciences, #347690, Lot Numbers: 4114575, 4288016)
anti-CD3/CD28-coated microbeads (Gibco, #11132D, Lot Numbers: 2811041, 3247935)
CD3-PerCP (SK7), CD4-FITC (SK3), CD8-PE (SK1) (BD Tritest, BD #342445, Lot Number: 25062)
anti-CD3-PerCP (BD Biosciences #345766, clone SK7, Lot number: 6012684)
anti-CD3-APC-H7 (BD #560176, clone SK7, Lot Number: 1172491)
anti-CD4-Pacific Blue (BD #558116, RPA-T4, 8234554)
anti-CD4-PerCPCy5.5 (BD #332772, SK3, 5114945)
anti-CD4-FITC (BD #555346, RPA-T4, 3303851)
anti-CD8-Pacific Blue (BD #558207, RPA-T8, 0314023)
anti-CD14-PE-Cy7 (eBioscience #25-0149-42, 61D3, 2198620)
anti-CD19-PE-Cy7 (BD #557835, SJ25C1, 2027311)
anti-CD45-V500 (BD #560777, HI30, 3030390)
anti-CD45-FITC (eBioscience #11-0459-42, HI30, 2586020)
anti-CD31-PE (BD #555446, WM59, 4330544)

anti-CD45RO-APC (BD #340438, UCHL-1, 5212861)
anti-CD45RO-VioGreen (Miltenyi # 130-106-802, UCHL-1, 5160317091)
anti-CD62L-FITC (BD #555543, Dreg-56, 4063893)
anti-CD326-PE-Cy7 (BioLegend #324222, 9C4, 2185095)
anti-HLA-DR-PE (eBioscience #12-9956-42, LN3, E11469-1634)
anti-CCR5-APC (BD #556903, 2D7, 3320980).
anti-CD16-FITC (BD #555406, 3G8, 0049228)
anti-CD56-FITC (BioLegend #304604, MEM-188, B386026)
anti-CD107a-PerCP-Cy5.5 (BioLegend #328616, H4A3, B322214)
anti-IL-2-Brilliant Violet 510 (BD #563265, 5344.111, 3275555)
anti-IFNγ-APC (BD #554702, B27, 1047222)
anti-TNF-PE (BD #559321, Mab11, 1174991).
anti-CD326-PE-Cy7 (BioLegend #324222, 9C4, 2185095)
α-hCD20-hIgG1 (Rituximab-biosimilar) (Invivogen, clone: Rituximab (Anti-hCD20-hIgG1, kappa), cat#: hcd20-mab1, lot#: 6415-46-01)
α-CD107a BV421 (Biolegend, 1:100, clone: H4A3, cat#: 328626, lot#: B412187)
α-NKG2C APC (Miltenyi Biotec, 1:100, clone: REA205, cat#: 130-117-398, lot#: 5231006251)
α-NKG2A PE Vio770 (Miltenyi Biotec, 1:100, clone: REA110, cat#: 130-113-567, lot#: 5230905002)
α-CD16 BV785 (Biolegend, 1:100, clone: 3G8, cat#: 302046, lot#: B368141)
α-Siglec7 APC Vio770 (Miltenyi Biotec, 1:25, clone: REA214, cat#: 130-101-009, lot#: 5231201070)
α-CD7 PerCP Cy5.5 (Biolegend, 1:100, clone: 4H9/CD7, cat#: 395602, lot#, B393466)
α-CD56 BUV395 (BD Bioscience, 1:100, clone: NCAM16.2, cat#: 563554, lot#: 4017789)
α-KIR3DL1 AF700 (Biolegend, 1:100, clone: DX9, cat# 312712, B333664)
α-KIR2DL2/DL3 BV711 (BD Bioscience, 1:50, clone: DX27, cat#: 745442, lot#: 0308852)
α-KIR2DL1 PE (Biolegend, 1:50, clone: HP-DM1, cat#: 374904, lot#: B402667)
α-CD57 PE Dazzle 594 (Biolegend, 1:100, clone: HNK-1, cat#: 359620, lot#: B227700)
α-CD2 AF700 (Biolegend, 1:100, clone: RPA-2.100, cat#: 300238, lot#: B365385)
α-ILT2 biotin (Miltenyi Biotec, 1:50, clone: REA998, cat#: 130-116-623, lot#: l5231201026)
α-CD161 BV605 (Biolegend, 1:100, clone: HP-3G10, cat#: 339916, lot#: B393009)
α-CD8 BV570 (Biolegend, 1:100, clone: RPA-T8, cat#: 301038, lot#: B346256)
α-CD14 BV510 (Biolegend, 1:50, clone: M5E2, cat#: 301842, lot#: B306943)
α-CD19 BV510 (Biolegend, 1:50, clone: HIB19, cat#: 302242, lot#: B399735)
α-CD57 pacific blue (Biolegend, 1:100, clone: HNK-1, cat#: 359608, lot#: B384454)
α-CD56 BUV737 (BD Bioscience, 1:100, clone: NCAM16.2, cat#: 612766, lot#: 6217711)
α-CD3 BUV395 (BD Bioscience, 1:100, clone: UCHT1, cat#: 563546, lot#: 3072678)
α-FcεR1γ FITC (Merck, 1:50, polyclonal, cat#: FCABS400F, lot#: 4003345)
α-PLZF PE (eBioscience, 1:100, clone: Mags.21F7, cat#: 12-9320-82, lot#: 2647691)
α-KIR3DL1 FITC (Biolegend, 1:100, clone: DX9, cat#: 312706, lo#: B365222)
α-KIR2DL1 APC Vio770 (Miltenyi Biotec, 1:25, clone: REA284, cat#: 130-118-345, lot#: 52408022198)
α-CD3 AF700 (BD Bioscience, 1:100, clone: UCHT1, cat#: 557943, lot#: 3263589)
α-CD56 BV786 (BD Bioscience, 1:100, clone: NCAM16.2, cat#: 564058, lot#: 2164817)
α-KIR2DL2/DL3 biotin (Miltenyi Biotec, 1:10, clone: DX27, cat#, 130-100-127, lot#: 5231103138)
α-NKG2A PE Cy7 (Beckman coulter, 1:100, clone: Z199, cat#: B10246, lot# 200081)
α-KIR3DL1/DS1 PE (Miltenyi Biotec, 1:50, clone: REA168, cat#: 130-125-972, lot#: 5231103122)
Streptavidin (BioLegend, 1:200, cat#: 405241, lot#: B373721)
α-CD16 BV711 (Biolegend, 1:100, clone: 3G8, cat#: 302044, lot#: B427826)
LIVE/DEAD™ Fixable Near-IR Dead Cell Stain Kit (Invitrogen, 1:1000, cat#: L34976, lot#:3079915)
Zombie Aqua™ Fixable Viability Kit (Biolegend, 1:50, cat# : 77143 lot: B291214)
Fluorescent cell tracer dye CFSE (Invitrogen, 1:2000, cat#: C34554, lot: 2486625)

Validation

All antibodies are commercially available and are validated for flow cytometry by the vendor on their official websites. Optimal antibody concentrations were determined by titration and calculation of the stain index. Positive and negative controls were included in all antibody experiments, with positive controls using antigen-containing plasma samples and negative controls using antigen-free plasma. Flourescenece-Minus-One controls were utilized to assess background fluorescence. Suitablity of antibody clones for the analysis of collagenase-treated cells was tested by subjecting PBMCs to collagenase digestion and comparing the staining patterns of treated and untreated cells. Specificity of the peroxidase conjugated Anti-Human IgG is determined by enzyme linked immunosorbent assay (ELISA). The conjugate is specific for human IgG when tested against human IgA, IgG, IgM, Bence Jones Kappa and Lambda myeloma proteins. Identity and purity of the antibody is established by immunoelectrophoresis (IEP), prior to conjugation. Electrophoresis of the antibody preparation followed by diffusion versus anti-goat IgG and anti-goat whole serum results in single arcs of precipitation. Anti-HIV-1 Env antibodies were tested against the 12 virus global panel to ensure their activity.

Eukaryotic cell lines

Policy information about cell lines and Sex and Gender in Research

Cell line source(s)

TZM-bl cells (NIH AIDS Reagent Program, Cat#8129). HEK293T (American Type Culture Collection). The sex of the TZM-bl and HEK293T cell lines is female. Raji cells provided by Ragon Institute of MGH, MIT and Harvard, Cambridge, MA, USA (RRID:CVCL_0511).

Authentication

TZM-bl and HEK293T cells were not authenticated.
Raji cells authentication was confirmed through morphology, expected behavior, and functionality.

Mycoplasma contamination

TZM-bl and HEK293T cells were not checked for Mycoplasma contamination.
Raji cells tested negative for mycoplasma contamination using the "Mycoplasma PCR Detection Kit" from abm (cat#: G238)

Commonly misidentified lines
(See ICLAC register)

No commonly misidentified cell lines were used in this study.

Plants

Seed stocks

n/a

Novel plant genotypes

n/a

Authentication

n/a

Flow Cytometry

Plots

Confirm that:

- [x] The axis labels state the marker and fluorochrome used (e.g. CD4-FITC).
- [x] The axis scales are clearly visible. Include numbers along axes only for bottom left plot of group (a 'group' is an analysis of identical markers).
- [x] All plots are contour plots with outliers or pseudocolor plots.
- [x] A numerical value for number of cells or percentage (with statistics) is provided.

Methodology

Sample preparation

Phenotyping was performed in heparinized whole blood immediately after sampling or on mucosal cells isolated by enzymatic digestion followed by Percoll gradient centrifugation. Functional analysis was performed in heparinized whole blood or on fresh PBMCs isolated by gradient density centrifugation. Cell sortings were performed from freshly isolated mucosal cells or from thawed PBMCs that were left to rest overnight at 37°C and 5% CO2 prior to processing.

Instrument

Cell analysis: BD FACSCanto II flow cytometer; BD LSRFortessa; Cytex Aurora 5 Laser
Cell sorting: BD FACSJazz; BD FACS Fusion

Software

Data collection: FACSDiva software v6.1.3 (BD Biosciences); BD FACS Sortware v1.1.0.84
Data analysis: FlowJo v10.10.0 and V10.8.1, R (v4.4.1), R Studio (v2024.04.0), Seumetry package (v0.1.0)

Cell population abundance

CD45+CD4+ cells were sorted from duodenal or ileal mucosal cells with the BD FACSJazz cell sorter. Post-sort fractions contained 170.000 cells and had a purity of over 98 % based on flow cytometric analysis. CD4+ T cells sorted from PBMCs using magnetic beads had a purity of over 95 % as determined by flow cytometric analysis.
KIR-panel (Fig. 4b + extended 6c): 60000-350000 NK cells were measured (B2: 60000 NK cells)
Adaptive NK cell panel (Fig. 4a+4c): 14000-70000 NK cells were measured (B2: 33000 NK cells)
Functional assay (Fig. 4d + extended 6d): 3000-4000 NK cells were measured.

Gating strategy

Lymphocytes were gated based on doublet discrimination and characteristic forward and side scatter properties. CD8+ or CD4+ T cells were identified by selecting cells that were negative for CD14 and CD19 and positive for CD3 and either CD8 or CD4. In degranulation assays, cells positive for CD16 and CD56 were additionally excluded. To determine the composition of CD4+ T cells, naive CD4+ T cells were identified by CD62L expression on CD45RO- CD4+ T cells and differentiated into CD31+ recent thymic emigrants and CD31- central naive T cells. Central memory T cells were classified by co-expression of CD45RO and CD62L, and effector memory T cells were classified by lack of CD62L expression. Terminally differentiated T cells were identified by the lack of CD62L expression on CD45RO- CD4+ T cells.

All lmyphocytes were initially gated based on FSC-A/SSC-A. Subsequently, single cells were identified using SSC-H/SSC-A and FSC-H/FSC-A., respectively FSC-W/FSC-A. In the adaptive NK cell panel, T cells (CD3+) were then excluded followed by exclusion of B cells (CD19+), monocytes (CD14+) and dead cells. NK cells were first identified by their CD7 and CD56 expression and thereafter conventional NK cell gate was set using CD16 and CD56 expression (Fig.4a, 4c).
In the KIR-panel, the single cell gate was followed by exclusion of B cells, monocytes and dead cells . NK cells were identified by their lack of CD3 and expression of CD56 (Fig. 4b).
In the functional assays, lymphocytes were gated based on their properties in the SSC-H and FSA-H plot. Single cells were identified using FSA-A/FSC-H. CFSE+ Raji cells and dead NK cells were excluded. NK cells were identified by their expression of CD16 and CD56 (Fig. 4d).

- [x] Tick this box to confirm that a figure exemplifying the gating strategy is provided in the Supplementary Information.
